# Rivaroxaban attenuates neutrophil maturation in the bone marrow niche

**DOI:** 10.1007/s00395-023-01001-5

**Published:** 2023-08-14

**Authors:** R. Schneckmann, M. Döring, S. Gerfer, S. Gorressen, S. Heitmeier, C. Helten, A. Polzin, C. Jung, M. Kelm, A. C. Fender, U. Flögel, M. Grandoch

**Affiliations:** 1 Institute for Translational Pharmacology Düsseldorf, Medical Faculty, University Hospital of the Heinrich Heine University, Universitätsstr. 1, 40225 Düsseldorf, Germany; 2 Department of Cardiothoracic Surgery, Heart Center of the University Hospital of Cologne, Cologne, Germany; 3 Institute for Pharmacology Düsseldorf, Medical Faculty, University Hospital and Heinrich Heine University, Düsseldorf, Germany; 4 Research & Development Pharmaceuticals, Bayer AG, Acute Hospital Research, Wuppertal, Germany; 5 Department for Cardiology, Pneumology and Vascular Medicine, University Hospital and Heinrich Heine University, Düsseldorf, Germany; 6 CARID, Cardiovascular Research Institute Düsseldorf, Medical Faculty and University Hospital Düsseldorf, Heinrich Heine University, Düsseldorf, Germany; 7 Institute of Pharmacology, University Hospital, University Duisburg-Essen, Essen, Germany; 8 Experimental Cardiovascular Imaging, Institute for Molecular Cardiology, University Hospital and Heinrich Heine University, Düsseldorf, Germany

**Keywords:** Rivaroxaban, Factor Xa Inhibitor, Myocardial infarction, Myeloid cells, Bone marrow

## Abstract

Pharmacological inhibition of factor Xa by rivaroxaban has been shown to mediate cardioprotection and is frequently used in patients with, e.g., atrial fibrillation. Rivaroxaban's anti-inflammatory actions are well known, but the underlying mechanisms are still incompletely understood. To date, no study has focused on the effects of rivaroxaban on the bone marrow (BM), despite growing evidence that the BM and its activation are of major importance in the development/progression of cardiovascular disease. Thus, we examined the impact of rivaroxaban on BM composition under homeostatic conditions and in response to a major cardiovascular event. Rivaroxaban treatment of mice for 7 days markedly diminished mature leukocytes in the BM. While apoptosis of BM-derived mature myeloid leukocytes was unaffected, lineage-negative BM cells exhibited a differentiation arrest at the level of granulocyte–monocyte progenitors, specifically affecting neutrophil maturation via downregulation of the transcription factors *Spi1* and *Csfr1*. To assess whether this persists also in situations of increased leukocyte demand, mice were subjected to cardiac ischemia/reperfusion injury (I/R): 7 d pretreatment with rivaroxaban led to reduced cardiac inflammation 72 h after I/R and lowered circulating leukocyte numbers. However, BM myelopoiesis showed a rescue of the leukocyte differentiation arrest, indicating that rivaroxaban's inhibitory effects are restricted to homeostatic conditions and are mainly abolished during emergency hematopoiesis. In translation, ST-elevation MI patients treated with rivaroxaban also exhibited reduced circulating leukocyte numbers. In conclusion, we demonstrate that rivaroxaban attenuates neutrophil maturation in the BM, which may offer a therapeutic option to limit overshooting of the immune response after I/R.

**Supplementary Information:**

The online version contains supplementary material available at 10.1007/s00395-023-01001-5.

## Introduction

Acute atherothrombotic events like myocardial infarction (MI) are followed by a massive immune response and mobilization of immune cells from the sites of hematopoiesis to the ischemic target tissue. A first initial wave of immune cell infiltration is required for the removal of dead cells and debris, a second wave initiates the subsequent reparative phase, and a proper orchestration of these steps is crucial for the entire healing process (reviewed in [17, 54]) [8, 29]. Thus, unraveling the complex immune cell-mediated inflammatory networks that are required for appropriate healing will open new therapeutic perspectives to improve patient outcomes.

Numerous preclinical and clinical data have identified the bone marrow (BM) as a central organ essentially contributing to emergency hematopoiesis after MI [62]. Tissue injury or infection rapidly triggers myeloid cell release into the circulation (reviewed in [7]), via proliferation of hematopoietic stem and progenitor cells (HSPCs), as well as through structural changes within the hematopoietic niche itself [15]. Part of the hematopoietic program is attributed to sympathetic innervation. Adrenergic signaling is known to drive myeloid cell production in hematopoietically active organs such as BM and spleen, to blunt HSPC quiescence and decrease retention signals in the hematopoietic niche [14, 16, 45]. As a consequence, myeloid cells are mobilized to the blood and recruited to the affected tissue.

Inflammation and leukocytosis post-MI are independent predictors of upcoming cardiovascular events; accordingly, the therapeutic value of blunting inflammatory signaling has been extensively evaluated in this context [3]. Global anti-inflammatory strategies, however, showed yet no benefit in terms of infarct size post-MI (Controlled Level EVERolimus in Acute Coronary Syndromes, a randomized, multicenter, international, double-blind, placebo-controlled trial, CLEVER ACS) and may even compromise the critical reparative phase. Thus, novel approaches for the targeted modulation of inflammatory responses, which also encompass adaptive hematopoiesis, are critically required.

Rivaroxaban, a direct inhibitor of activated coagulation factor X (FXa), has been approved for several cardiovascular indications including the prevention and treatment of venous thromboembolism, stroke prophylaxis in nonvalvular atrial fibrillation, and to reduce the risk of major cardiovascular events in coronary heart or peripheral artery disease [1]. In patients with a recent acute coronary syndrome (ACS), treatment with rivaroxaban as an add-on to antiplatelet therapy has been associated with a significant reduction in the composite endpoint of death from cardiovascular events, such as MI and stroke [44]. Mechanistically, rivaroxaban is thought to protect against recurrent thromboembolic events by targeting FXa as the primary and rate-limiting source of amplification in the coagulation cascade [11]. Additional coagulation-independent anti-inflammatory actions of rivaroxaban have also been demonstrated in clinical [35, 37, 43] and preclinical contexts [19, 21, 30, 38, 41, 46], associated with anti-fibrotic [37] and plaque-stabilizing effects [27] in mouse models of atherosclerosis.

The direct cellular actions of FXa involve proteolytic cleavage of a family of protease-activated receptors (PAR). Proteolysis of a specific N-terminal domain unmasks a so-called tethered ligand sequence that self-activates the receptor, thereby stimulating G-protein activation and signaling [51]. Of the four known PAR, PAR1, most notably PAR2, and to a lesser extent PAR4, respond to FXa, and—given their abundant distribution in various cell types of the cardiovascular and immune system—are likely to mediate a plethora of coagulation-independent FXa effects. PARs are potent drivers for production of inflammatory mediators as well as cell proliferation and migration [64], and as such are intimately involved in wound healing, tissue repair, and remodeling. Rivaroxaban is therefore anticipated to exert beneficial cardiovascular effects beyond anticoagulation, by indirectly inhibiting FXa-triggered PAR activation (reviewed in [10, 64]). Especially signaling through PAR-1/2 is postulated to impact myocardial pathologies [2] and atherosclerosis [33], at least in part by modulating pro-inflammatory gene expression, macrophage polarization [12] and macrophage autophagy [32]. Of note, myeloid cells have also been described to themselves generate and secrete FX/FXa, with rivaroxaban reported to exhibit antitumor immunity by blocking the FXa–PAR-2 pathway, leading to the reprograming of tumor-associated macrophages [22, 48]. Whether similar mechanisms also contribute to the anti-inflammatory effects of rivaroxaban in the cardiovascular context is unknown.

The pathophysiological significance of the FXa–PAR-2 axis appears to extend beyond the local tissue level. PAR-2 deficiency in hematopoietic cells was notably shown to attenuate cardiac inflammation and fibrosis post-MI [71], implicating an important but so far underestimated role of PAR-2 in the context of emergency hematopoiesis. Coagulation factors have in fact been attributed a crucial role in the endosteal and vascular hematopoietic stem cell niche (reviewed in [47]), but a modulating impact of the FXa inhibitor rivaroxaban on emergency hematopoiesis is currently unknown. Also, the relative contribution of FXa compared to other locally produced or circulating factors that may also regulate PAR-2 expression and function is yet unclear.

Multiple pleiotropic effects of rivaroxaban are therefore anticipated in the context of ACS and MI. The coagulation-independent response in remote organs like the BM, however, are only poorly understood. In this study, we explored the impact of rivaroxaban on hematopoiesis and leukocyte dynamics under homeostatic conditions and in a model of acute MI.

## Methods

### STEMI patients

Blood sample collection in patients with ST-segment elevation was performed on the day of hospitalization (d0) and 4 days later (d4). To assess cardiac function, magnetic resonance imaging (MRI) was performed between day 4 and 6. Rivaroxaban treatment was initiated at the acute event in all patients and before the catheter laboratory and patients received either antiplatelet medicine alone (control) or in combination with standard dose rivaroxaban. One patient in the rivaroxaban group was pretreated with a vitamin-K antagonist before admission to the hospital.

All patient samples were obtained in accordance with the Declaration of Helsinki and after approval by the local Ethics Committee of the Heinrich Heine University Düsseldorf (STEMI-registry 2019–557 and DOSAR-registry 2018–48). All persons gave their informed consent prior to their inclusion in the study.

### Mice and drug administration

Male 10- to 12-week-old C57BL/6 J mice (Janvier Labs, Le Genest-Saint-Isle, France) were fed a standard laboratory diet supplemented with or without 1000 mg rivaroxaban per kg diet (Ssniff, Soest, Germany). Based on a pilot study in collaboration with Bayer HealthCare, Wuppertal, this dose was shown to produce clinically relevant plasma levels. In detail, mice were administered doses of 0.6 or 1.2 g of rivaroxaban per kilogram for 7 days prior to ischemia–reperfusion (I/R) injury. Rivaroxaban plasma concentrations were measured 24 h before and after cardiac I/R. Blood samples were collected at different time points, specifically at 9 AM and 9 PM. The corresponding data can be found in Supplementary Fig. 1 and Supplementary Table 1. Rivaroxaban was kindly provided by Bayer HealthCare, Wuppertal. Pretreatment started 7 days prior to induction of cardiac ischemia and reperfusion injury (I/R) and was continued until 72 h post-I/R. To avoid declining plasma levels during the surgery, mice received an additional dose of rivaroxaban [each 4 mg/kg body weight solved in Kollisolv^®^ and water (60:40)] or vehicle via oral gavage. Mice were kept on a 12 h’ dark/light cycle with access to diet and water ad libitum. Animal experiments were performed according to the German Animal Welfare Law and were authorized by the local Animal Ethics Committee (LANUV; State Agency for Nature, Environment and Consumer Protection). To avoid variations caused by fluctuating estrus cycle in female individuals, all experiments were performed in male mice. Mice were excluded from the experiment when certain criteria of suffering were observed. These included weight losses greater than 20% of body weight, cessation of food and water ingestion or lack of voluntary movement. Mice were allocated to randomization and all I/R experiments were performed in a blinded fashion.

### Ischemia reperfusion injury (I/R)

Cardiac ischemia and reperfusion injury was induced by ligation of the left ascending coronary artery (LAD). In brief, mice were anesthetized by intraperitoneal injection of ketamine (90 mg/kg, Pfizer) and xylazine (15 mg/kg, Bayer), followed by intubation and connection to a rodent ventilator (Uno Micro Ventilator, Uno B.V., Zevenaar, The Netherlands). To maintain anesthesia, mice were respirated with a tidal volume of 10 µl/g BW at a rate of 140 strokes/min using a mixture of 2/3 air, 1/3 oxygen and 2.0% isoflurane (Forene^®^, Abbott GmbH, Germany). During surgery, body temperature was maintained at 37 °C while continuously monitoring the mice's electrocardiogram (ECG) (Hugo Sachs Elektronik—Harvard Apparatus GmbH, March-Hugstetten, Germany). After left lateral thoracotomy between the third and fourth rib, the pericardium was incised to place a 7-0 surgical suture connected to a PE-10 tubing around the LAD approximately 1 mm distal from the tip of the left auricle. Temporal ischemia was induced by suture occlusion. The correct position of the suture was confirmed by blanching of the apex and ST-elevation in ECG. After 45 min of ischemia, suture was cut to allow the heart to reperfuse. The chest was closed with 4-0 and 5-0 suture. After signs of spontaneous breathing, mice were extubated. Postoperative analgesia was achieved by subcutaneous injection of buprenorphine (0.05 mg/kg, Temgesic, Essex Pharma GmbH, Munich, Germany). Mice were killed after 24 or 72 h post-I/R.

### 2,3,5-Triphenyltetrazolium chloride (TTC) staining

After 24 h of reperfusion the animals were killed and the heart was explanted for IS measurements. The heart was rinsed in 0.9% normal saline, the LAD was re-occluded, and 4% Evans Blue dye was injected into the aortic root to delineate the area at risk (AAR) from not-at-risk myocardium. The tissue was wrapped in a clear cool wrap and stored for 1 h in a − 20 °C freezer. The heart was then serially sectioned parallel to the atrio-ventricular groove in 1-mm slices, and each slice was weighed. Viable and necrotic sections of the AAR were identified by incubating the heart in 1% 2,3,5-triphenyltetrazolium chloride for 5 min at 37 °C. The areas of infarction, AAR, and non-ischemic LV were assessed with computer-assisted planimetry (Diskus software; Hilgers, Königswinter, Germany) by an observer blinded to the sample identity. The size of the myocardial infarction was determined by the following equation: (A1 × Wt1) + (A2 × Wt2) + (A3 × Wt3) + (A4 × Wt4) + (A5 × Wt5) + (A6 × Wt6), where *A* is percent area of infarction by planimetry from subscripted numbers 1–6 representing sections and Wt is the weight of the same numbered sections.

### Cardiac echocardiography

Cardiac images were acquired using a Vevo 3100 high-resolution ultrasound scanner with 20–46 MHz linear transducer (MX400, VisualSonics Inc.) pre-ischemia, 72 h, and 7 d post-ischemia (as described in [28]). Echocardiography was performed under slight mask anesthesia by an inhaled mixture of 1.5–2.0% isoflurane and 100% oxygen. ECGs were obtained with built-in ECG electrode contact pads. Body temperature was maintained at 36.5–37.5 °C by a heating pad and infrared lamp. All hair was removed from the chest using a chemical hair remover (Veet). Aquasonic 100 gel (Parker Laboratories, Hellendoorn, The Netherlands) was applied to the thorax surface to optimize the visibility of the cardiac chambers. Parasternal long- and short-axis views were acquired. Left ventricular (LV) end-systolic and end-diastolic volumes (ESV and EDV) were calculated using the bi-plane Simpson method. Simpson method is based on the diastolic and systolic lengths of the ventricle obtained from PSLAX B-mode and the endocardial tracing of the three SAX B-modes (mid-ventricular, apical, basal) at both diastole and systole (*V* = (area mid-ventricular + area apical + area basal) * h/3, where *h* = ventricular length). Ejection fraction was calculated using the formula EF = (EDV − ESV)/ EDV*100. A single ultrasound session ranged from 15 to 20 min per mouse.

### Magnetic resonance Imaging (MRI)

Data were recorded 21 days after induction of I/R at a Bruker AVANCE^III^ 9.4 T wide bore NMR spectrometer driven by ParaVision 5.1 (Bruker, Rheinstetten, Germany). Images were acquired using a Bruker microimaging unit Micro2.5 with actively shielded gradient sets (1.5 T/m) and a 25 mm quadrature resonator (Bruker) optimized for cardiovascular applications essentially as previously described [9, 56]. Mice were anesthetized with 1.5% isoflurane and kept at 37 °C. The front paws and the left hind paw were attached to ECG electrodes (Klear-Trace; CAS Medical Systems, Branford, CT, USA) and respiration was monitored by means of a pneumatic pillow positioned at the animal’s back. Vital functions were acquired by a M1025 system (SA Instruments, Stony Brook, NY, USA) and used to synchronize data acquisition with cardiac and respiratory motion. For functional analysis, high-resolution images of mouse hearts were acquired in short-axis orientation using an ECG- and respiratory-gated segmented fast gradient echo cine sequence with steady state precession (FISP). A flip angle (FA) of 15°, echo time (TE) of 1.2 ms, 128 segments, and a repetition time (TR) of about 6–8 ms (depending on the heart rate) were used to acquire 16 frames per heart cycle; field of view (FOV), 30 × 30 mm^2^; matrix 256 × 256, slice thickness (ST), 1 mm; number of averages (NA), 2; zero-fill acceleration (ZFA), 2; acquisition time (TAcq) per slice for one cine loop, ~ 1 min. Routinely, eight to ten contiguous short-axis slices were required for complete coverage of the LV. For evaluation of functional parameters (e.g. EDV and ESV, ejection fraction (EF) etc.), ventricular demarcations in end-diastole and -systole were manually drawn with the ParaVision Region-of-Interest (ROI) tool.

### Flow cytometry

For flow cytometric analyses, the organs of 10- to 12-week-old mice were harvested after 7 days of treatment or 72 h post-I/R. Single cell suspensions of blood, spleen, and heart were obtained as previously described [23, 50].

BM cells were obtained by flushing the femora and tibiae with ice-cold PBS. Cell suspensions were meshed through a 70 µM filter. After centrifugation at 300*g* for 10 min at 4 °C, erythrocytes were lysed with hypotonic ammonium chloride solution. Before staining with the antibodies depicted in Supplementary Tables 2, 3, cells were resuspended in PEB (PBS containing 2 mM EDTA and 0.5% bovine serum albumin (BSA)).

Absolute cell concentrations were determined with flow-count fluorospheres (Beckman Coulter Inc., Krefeld, Germany). Flow cytometric measurements were performed with an LSRII flow cytometer (Becton-Dickinson, Heidelberg, Germany) and Kaluza Flow Analysis Software (Beckman Coulter Inc.) or FlowJo Software (Treestar, San Carlos, CA) were used for subsequent data analysis. After discrimination of single cells, living cells were selected for further gating.

Cardiac leukocytes were identified as CD45^+^ cells, cardiac myeloid leukocytes as CD45^+^CD11b^+^, cardiac neutrophils as CD45^+^CD11b^+^Ly6G^+^, and cardiac macrophages as CD45^+^CD11b^+^Ly6G^−^CD11c^−^F4/80^+^. Blood monocytes were identified as CD11b^+^CD115^+^ cells and blood neutrophils as CD11b^+^Ly6G^+^ cells. BM myeloid leukocytes were identified as CD11b^+^ cells, BM neutrophils as CD11b^+^Ly6G^+^ cells, and BM monocytes as CD11b^+^CD115^+^ cells. Splenic myeloid leukocytes were identified as CD11b^+^ cells, splenic neutrophils as CD11b^+^Ly6G^+^ cells, splenic monocytes as CD11b^+^CD115^+^ cells, and the subsets of Ly6C high monocytes as CD11b^+^CD115^+^Ly6C^high^, Ly6C intermediate monocytes as CD11b^+^CD115^+^Ly6C^int^, and Ly6C low monocytes as CD11b^+^CD115^+^Ly6C^low^.

Myeloid progenitors (MPs) were identified as lineage^−^cKit^+^Sca1^−^ cells, CMPs were identified as lineage^−^cKit^+^Sca1-CD34^+^CD16/32^−^ cells, GMPs as lineage^−^cKit^+^Sca1^−^CD34^+^CD16/32^+^ cells, MEPs as lineage^−^cKit^+^Sca1^−^CD34^−^CD16/32^−^ cells, LSKs as lineage^−^cKit^+^Sca1^+^CD34^+^CD16/32^−^ cells, MPPs as lineage^−^cKit^+^Sca1^+^CD127^−^CD34^+^CD135^+^, LT-HSC as lineage^−^cKit^+^Sca1^+^CD127^−^CD34^−^CD135^−^, ST-HSC as lineage^−^cKit^+^Sca1^+^CD127^−^CD34^−^CD135^−^, Ly6C + GMPs as cKit^+^Sca1^−^CD34^+^CD16/32^+^Ly6C^+^, common monocyte progenitors (cMoPs) as cKit^+^Sca1^−^CD34^+^CD16/32^+^Ly6C^+^CD135^−^CD115^+^, proNeutrophiles (proNeus) as cKit^+^Sca1^−^CD34^+^CD16/32^+^Ly6C^+^CD135^−^CD115^−^, proNeus1 as cKit^+^Sca1^−^CD34^+^CD16/32^+^Ly6C^+^CD135^−^CD115^−^CD11b^low^CD106^−^, and proNeus2 as cKit^+^Sca1^−^CD34^+^CD16/32^+^Ly6C^+^CD135^−^CD115^−^CD11b^high^CD106^+^.

Representative gating schemes are given in Supplementary Figs. S2, S3, and S4. Fluorescence minus one (FMO) and isotype controls were used to determine the respective gates.

### Proliferation of hematopoietic stem and progenitor cells from the BM

To study the proliferation of HSPCs, mice were injected with 10 mg/ml 5-brom-2′-deoxyuridin (BrdU, Sigma Aldrich, # B9285-250MG) as early as 1 hour before harvest. BM cells were obtained and prepared for flow cytometric analysis as described above. To analyze the proportion of BrdU in the cell populations, and therefore proliferating cells, further staining was performed after the primary antibody staining. Since BrdU is incorporated into DNA and is therefore an intracellular marker, cells were fixed and permeabilized using a Fix & PERM^™^ cell permeabilization kit (Invitrogen, #GAS004). The cells were then treated with deoxyribonuclease (DNase) to release the BrdU incorporated into the DNA. Cells were then stained with PE anti-BrdU antibody (Biolegend, San Diego, USA, #364116). Proliferating cells were identified as BrdU positive (BrdU^+^).

### Apoptosis in myeloid leukocytes in cardiac blood and BM

Annexin V staining was used to determine the proportion of apoptotic cell populations. BM and blood cells were collected and prepared for flow cytometric analysis as described above. After primary antibody staining, cells were stained with PerCP/Cyanine5.5 Annexin V (Biolegend, San Diego, USA, #640936) using Annexin V binding buffer. Apoptotic cells were identified as Annexin positive (Annexin^+^).

### Isolation of lineage^+^, lineage^−^, and c-Kit^+^ cells from the bone marrow

Lineage^+^, Lineage^−^ and c-Kit^+^ BM-derived cells were isolated by immunomagnetic bead separation. After flushing femora und tibiae from 10 to 12 weeks old C57BL/6 J mice with ice cold PBS, BM aspirates were collected and meshed through a 70 μm filter. Cells were enriched using a Lineage Cell Depletion Kit (#130-090-858, Miltenyi Biotec, Bergisch Gladbach, Germany) or CD117 MicroBeads (130-091-224 Miltenyi Biotec, Bergisch Gladbach, Germany) according to the instruction manual. Lineage-depleted cells were further processed for ex vivo differentiation into monocytes/macrophages or neutrophils. Lineage^+^ cells were incubated with hyperosmotic buffer to lyse erythrocytes and cultivated further to assess apoptosis. C-Kit ^+^ cells were aspirated in TRIzol and further processed for quantitative real-time polymerase chain reaction (qPCR) analysis.

### Cytokine-induced differentiation of lineage-depleted cells

Lineage^−^ cells were differentiated into myeloid cells according to a previously described protocol with slight modifications [41]. In brief, cells were cultivated in Gibco Roswell Park Memorial Institute (RPMI) 1640 Medium + 20% heat-inactivated fetal calf serum (FCS), and 1% penicillin–streptomycin (PS) supplemented with 10 μM rivaroxaban or 0.1% DMSO (vehicle control) during the entire differentiation process. During all medium changes, cells were centrifuged and washed intensively with sterile PBS. Differentiation was induced by addition of specific cytokine cocktails (all provided by Stem Cell Technologies, Vancouver, Canada) as follows.

Cells were incubated with 50 ng/ml stem cell factor (SCF) and 50 ng/ml interleukin (IL)-3 for 3 days to induce myeloid progenitor differentiation. To avoid growth arrest due to increased cell density, cells were split after 2 days. On day 3, cells were divided and differentiated into either monocytes or neutrophils. For the generation of monocytes, cells were incubated with 50 ng/ml each of granulocyte–monocyte colony-stimulating factor (GM-CSF) and macrophage colony-stimulating factor (M-CSF) for 2 days. For the generation of neutrophils, cells were incubated with 50 ng/ml each of IL-3, SCF, and granulocyte colony-stimulating factor (G-CSF) until day 5, followed by stimulation with 50 ng/ml G-CSF up to day 7. To study the impact of FXa inhibition, differentiation was carried out in the presence of either rivaroxaban (BAY 59–7939, 10 µM, Selleckchem, # S3002) or apixaban (BMS 562247–01, 10 µM, Selleckchem, # S1593). To elucidate the involvement of PAR receptors, differentiation medium supplemented with specific antagonists against PAR1 (voraxapar; 10 µM, dissolved in DMSO, Selleckchem, #S8067), PAR2 (AZ3451; 10 µM, Sigma Aldrich, # SML2050) or PAR4 (ML354; 200 nM, Merck, # SML1439), rivaroxaban, or DMSO vehicle (0.1% DMSO) was applied 15 min before adding 30 nmol/ml FXa (Merck).

At the indicated time points, cells were snap frozen in peqGOLD TriFast™ (Peqlab, Erlangen, Germany) for RNA isolation or harvested for flow cytometry. Giemsa staining (Merck, Darmstadt, Germany) of cell smears was performed to determine cell morphology.

Antibodies used for flow cytometry of the differentiation process are depicted in Supplementary Table 4. Differentiation was assessed by loss of hematopoietic stem and progenitor marker and gain of lineage marker as well as myeloid cell marker. Representative Giemsa stains showing the different cell morphologies at distinct time points of differentiation as well as the loss of stem cell markers and gain of mature immune cell markers as measured by flow cytometry are shown in Supplementary Figs. 5, 6, 7. BM-derived myeloid leukocytes were identified as CD45^+^CD11b^+^ cells, BM-derived neutrophils as CD45^+^CD11b^+^Ly6G^+^ cells, and BM-derived CD115^+^ monocytes as CD45^+^CD11b^+^CD115^+^ cells. Myeloid progenitors (MPs) were identified as lineage^−^cKIT^+^Sca1^−^ cells, CMPs were identified as lineage^−^cKIT^+^Sca1^−^CD34^+^CD16/32^−^ cells, GMPs as lineage^−^cKIT^+^Sca1^−^CD34^+^CD16/32^+^ cells, and MEPs as lineage^−^cKIT^+^Sca1^−^CD34^−^CD16/32^−^ cells. Representative gating schemes are given in Supplementary Figs. 8, 9.

### Apoptosis of lineage-positive (lineage^+^) cells

Lineage^+^ cells derived from BM were cultivated with RPMI + 10% heat-inactivated FCS + 1% PS supplemented with 10 μM rivaroxaban or DMSO (0.1%, vehicle control). After 24 h, cells were collected, centrifuged, and washed with sterile PBS. Cell pellets were diluted in PBS supplemented with 0.5% bovine serum albumin and 5 mM EDTA. To avoid unspecific binding, cells were incubated with a purified anti-mouse CD16/32 antibody prior to incubation with a Live/Dead (LD) dye. After staining with antibodies against mature leukocytes, cells were washed intensively with Annexin binding buffer. To assess apoptosis, cells were incubated with PerCP/Cyanine5.5 Annexin V (Biolegend, San Diego, USA). Non-apoptotic cells were identified as LD^−^Annexin^−^ cells, early apoptotic cells as LD^−^Annexin^+^ cells, and late apoptotic cells as LD^+^Annexin^+^ cells.

### RNA isolation and cDNA synthesis

RNA was isolated from hearts and BM-derived cells. To this end, mice were killed, hearts were excised, and the left ventricle was dissected free of septum and right ventricle. Tissues were snap frozen in liquid nitrogen and stored at − 80 °C until further processing. For RNA analysis, tissue samples and cells were homogenized with 1 mL of peqGOLD TriFast^™^ (Peqlab, Erlangen, Germany) using the gentleMACS Dissociator and isolated by chloroform–phenol extraction. RNA concentration and purity (260 nm/280 nm quotient) were determined by a spectrometer (Nanodrop, Peqlab, Erlangen). cDNA was synthesized using the QuantiTect^®^ Reverse Transcription Kit (Qiagen, Erkrath, Germany, #205311). For cDNA synthesis, 1 µg of left ventricular or cellular RNA was used.

### Quantitative real-time polymerase chain reaction (qPCR)

qPCR experiments were performed using Platinum^®^ SYBR^®^ Green qPCR SuperMix-UDG (Life Technologies, USA, #11733038) with ROX reference dye on the StepOnePlus^™^ Real-Time PCR System (Life Technologies, Singapore, Singapore). Samples were measured in duplicate and analyzed by the ΔΔCT method. Primers used are depicted in Supplementary Table 4.

### Statistical analysis

Sample size estimation was based on previous results in comparable studies, assuming 80% power at a significance level of 0.05. Specific details on how many independent biological samples or mice were included in an experiment or how many times the experiments were repeated independently are given in the corresponding figure legends. Sample size is given in the figure legends and data are presented as mean ± standard deviation (SD) or symbols and lines. Categorical variables are expressed as percentage (number). For continuous variables, normality and homogeneity of variance were assessed by Shapiro–Wilk and Brown–Forsythe tests, respectively. After confirming homogeneous variances and normality, two-group comparisons for means were performed by two-sided unpaired Student’s *t* test. In case of inhomogeneous variances, Mann–Whitney* U* test was performed. For comparison of two dependent groups with normally distributed matched values, paired *t* test was used. If normal distribution was not given, the Wilcoxon-matched signed rank test was used. Multi-group comparisons for dependent groups were performed using mixed-effects analysis, followed by Sidak post hoc test. Multi-group comparisons for means were performed by one-way analysis of variance (ANOVA), followed by Tukey multiple comparison test. In case of inhomogeneous variances, Kruskal–Wallis, followed by Dunn’s test were performed. The Chi-square test or Fisher exact test was used to analyze differences between categorical variables. *P* < 0.05 was considered statistically significant. Statistical analyses mentioned above were performed using GraphPad Prism version 9.3.0 and SPSS Statistics version 25.

## Results

### Factor Xa inhibition alters BM composition in mice

**Fig. 1:**
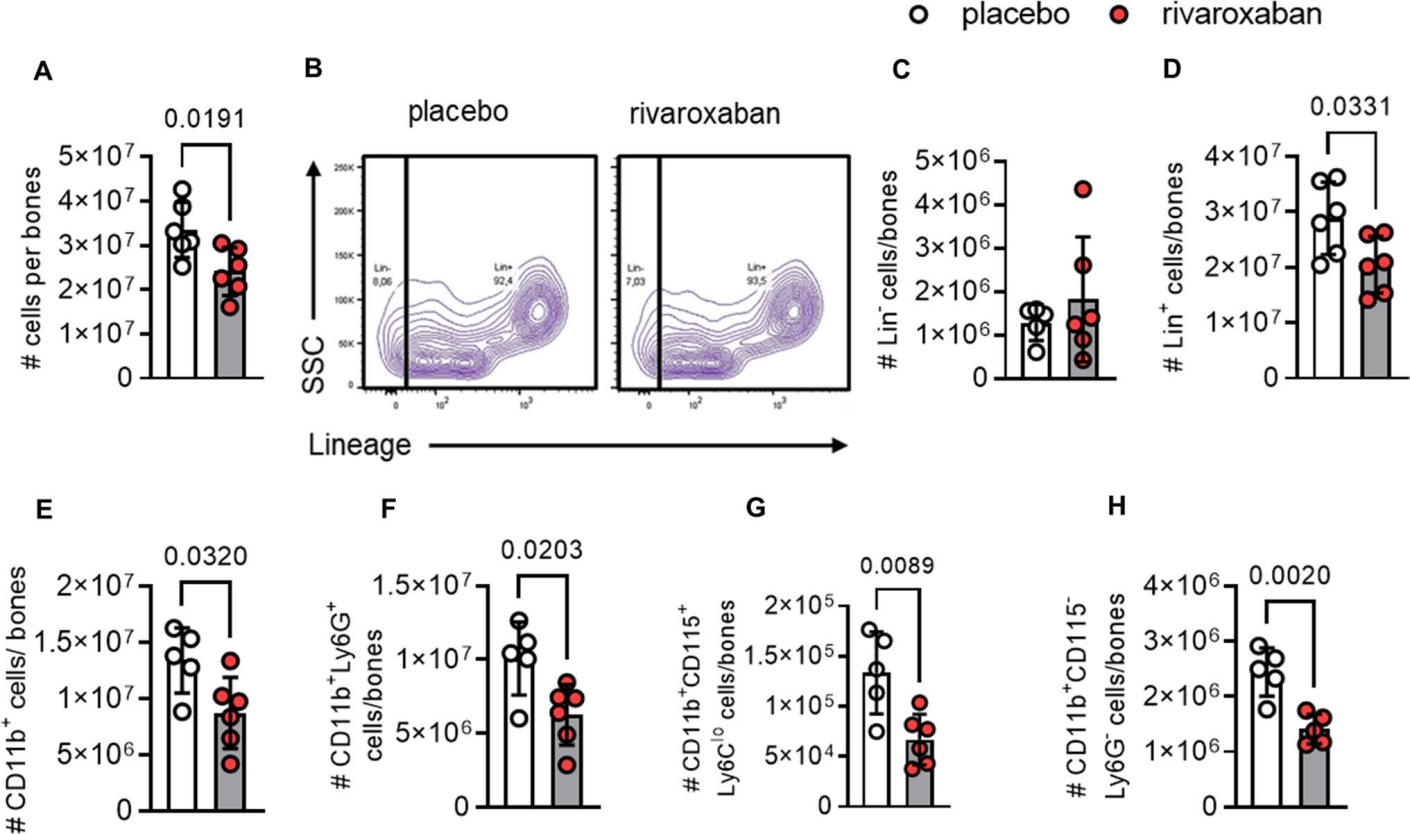
Decreased myeloid cell numbers in the bone marrow of rivaroxaban-treated mice. C57BL6/J mice were treated with placebo or rivaroxaban for 7 days, followed by flow cytometry of the bone marrow (BM) from the tibiae and femora. **A** Total cells. **B** Flow plot and quantification of **C** lineage-negative (Lin^−^) and **D** lineage-positive (Lin^+^) cells. Quantification of **E** myeloid leukocytes (CD11b^+^), **F** neutrophils (CD11b^+^Ly6G^+^), **G**, Ly6C^lo^ monocytes (CD11b^+^CD115^+^Ly6C^lo^) and **H** CD115^−^Ly6G^−^ myeloid leukocytes (CD11b^+^CD115^−^Ly6G^−^) shown as absolute cell numbers. Data represent mean ± SD, *P < 0.05, **P < 0.01*.* Statistical significance was determined by unpaired *t* test. SSC indicates side scatter

In a first step, we established the treatment dose for the FXa inhibitor rivaroxaban in our mice similar to recent publications [32] and representing standard dose rather than low dose in humans (Supplementary Fig. 1 and Supplementary Table 1). For evaluating rivaroxaban's effects on the bone marrow, mice were fed a standard laboratory diet supplemented with 1000 mg rivaroxaban per kg diet for 1 week. Flow cytometric analyses revealed reduced BM cellularity (2.4 × 10^7^ cells) of rivaroxaban-treated mice compared to the respective controls (3.3 × 10^7^ cells) (Fig. 1A). While the absolute number of lineage-negative cells (Lin^−^) composed of hematopoietic stem and progenitor cells (HSPC) and niche cells was unaffected (Fig. 1B–C), rivaroxaban suppressed Lin^+^ mature leukocytes (Fig. 1B, D). Further analysis revealed a significant reduction of CD11b^+^ myeloid leukocytes (Fig. 1E), neutrophils (Fig. 1F), Ly6C^lo^ monocytes (Fig. 1G), and CD115^−^Ly6G^−^ myeloid cells (Fig. 1H). Of note, no difference was seen in the absolute numbers of the myeloid precursors megakaryocyte–erythrocyte progenitors (MEPs; Supplementary Fig. S10A, B), common myeloid progenitors (CMPs; Supplementary Fig. S10C,D), or hematopoietic stem cells (HSCs; Supplementary Fig. 10F-M) as well as in the amount of lymphocytes (Supplementary Fig. 11).

To verify whether BM hypocellularity resulted from decreased myeloid cell survival in response to rivaroxaban, we measured apoptosis by flow cytometry (Supplementary Fig. 12). After isolation of BM-derived leukocytes via immunomagnetic bead separation, cells were cultivated with or without rivaroxaban for 24 h (Supplementary Fig. 12A). By using combined Annexin V/Live Dead dye staining, we determined the percentage of non-apoptotic, early apoptotic, and late apoptotic cells. Since FXa inhibition did not enhance apoptosis of CD11b^+^ myeloid leukocytes (Supplementary Fig. 12B–C), neutrophils (Supplementary Fig. 12D–E), or CD115^−^Ly6G^−^ myeloid cells (Supplementary Fig. 12F–G), increased apoptosis is unlikely to explain the reduced myeloid cell abundance in the BM of rivaroxaban-treated mice. Next, we therefore explored the potential effects of FXa inhibition on myelopoiesis.

### Rivaroxaban inhibits differentiation in granulocyte–monocyte progenitors in vitro

**Fig. 2:**
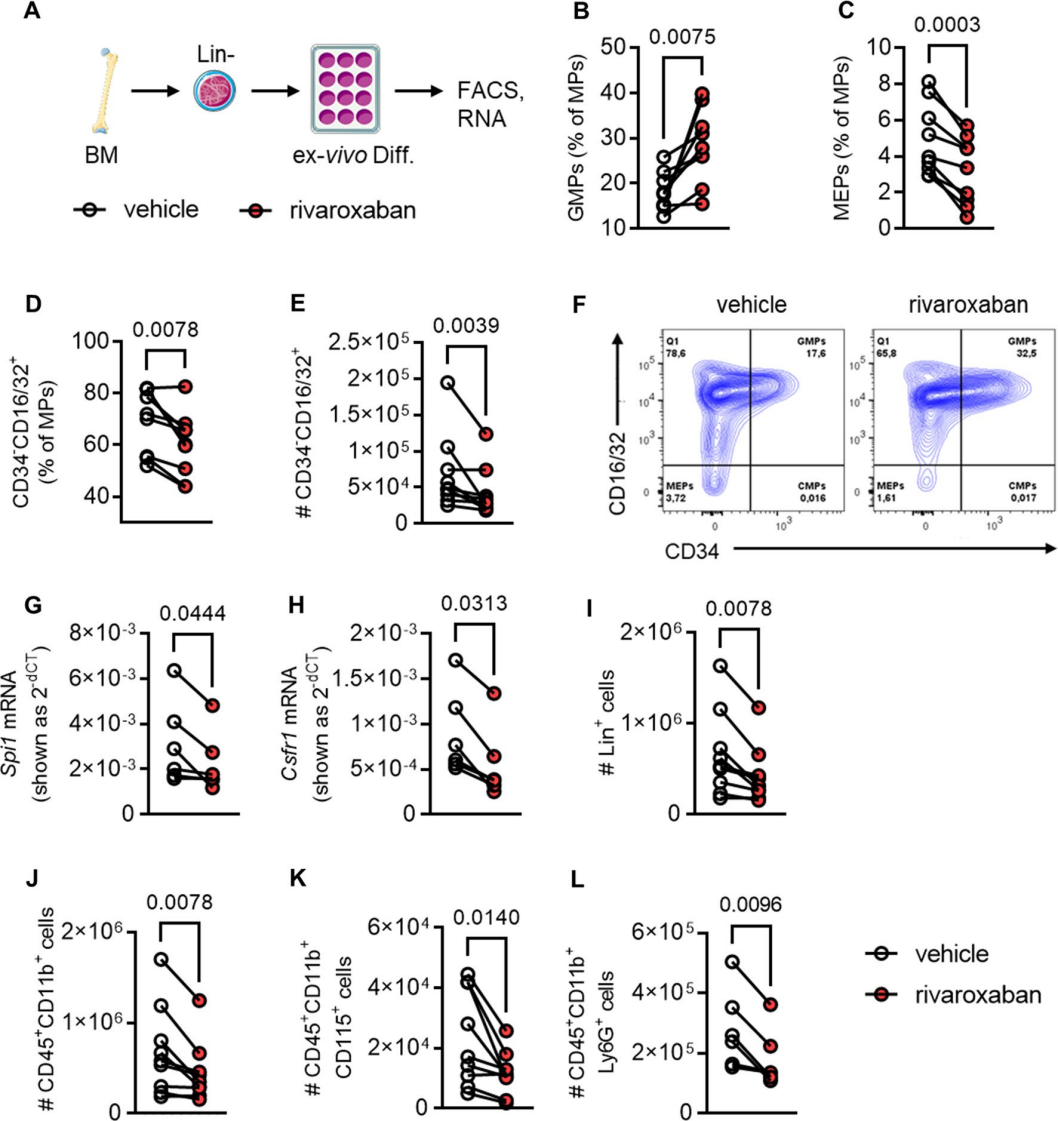
Rivaroxaban leads to differentiation arrest in granulocyte–monocyte progenitors during myelopoeisis by downregulation of *Spi1* (PU.1) and *Csf1r* expression. **A** Experimental outline showing ex vivo differentiation of bone marrow-derived (Lin)eage^−^ cells into myeloid progenitors (MPs) (d3) and monocytes (d5). Cells were treated with vehicle or 10 μM rivaroxaban and harvested for flow cytometric analyses or RNA isolation followed by semiquantitative *realtime* qPCR. Quantification of **B**, granulocyte–monocyte progenitors (GMPs; Lin^−^Sca1^−^c-KIT^+^CD34^+^CD16/32^+^), **C** megakaryocyte–erythrocyte progenitor cells (MEPs; Lin^−^Sca1^−^c-KIT^+^CD34^−^CD16/32^−^), and **D** CD34^−^CD16/32^+^ cells (Lin^−^Sca1^−^c-KIT^+^CD34^−^CD16/32^+^) shown as percentages of MPs and **E** absolute numbers of (#) CD34^−^CD16/32^−^ cells after 3 days of differentiation. **F** Flow plot of MPs. mRNA expression of **G** spleen focus forming virus (SFFV) proviral integration oncogene (*Spi1)* and **H** colony-stimulating factor 1 receptor (*Csfr1).* Quantification of **I** Lin^+^ cells, **J** myeloid leukocytes (CD45^+^CD11b^+^), **K** CD115^+^ monocytes (CD45^+^CD11b^+^CD115^+^) and **L** neutrophils (CD45^+^CD11b^+^Ly6G^+^) shown as absolute cell numbers after 5 days of differentiation, *n* = 6–9. Data represent symbols and lines. *P < 0.05, **P < 0.01, ***P < 0.001. Paired *t* test **(B**–**C**, **G**, **K)** and Wilcoxon matched-pairs signed rank test **(D**–**E**, **H**–**J, L)**. # indicates absolute cell numbers

To investigate the impact of rivaroxaban during different stages of myelopoiesis, we analyzed cytokine-induced neutrophil and monocyte differentiation [24] in detail (Fig. 2A). Flow cytometry performed after 3 days of differentiation, highlighted a significant decrease in the percentage of MEPs in rivaroxaban-treated cultures (Fig. 2C, F). More importantly, we detected a strong increase in GMPs (Fig. 2B, F) and decrease of CD34^−^CD16/32^+^ cells (Fig. 2D–F). Hypothesizing that altered progenitor cell composition might result either from increased MEP and CD34^−^CD16/32^+^ cell apoptosis or improved survival of GMPs, we measured apoptosis by Annexin V staining after 24 or 72 h (Supplementary Fig. 13) of differentiation. Unexpectedly, at the indicated time points, neither MEP and CD34^−^CD16/32^+^ cell apoptosis was increased nor was GMP apoptosis decreased.

Since mature leukocytes express the FC receptors CD16/32 but lose the progenitor marker CD34 during differentiation, we hypothesized that rivaroxaban induced differentiation arrest in GMPs. Indeed, we observed decreased numbers of Lin^+^ cells **(**Fig. 2I**)**, CD11b^+^ myeloid leukocytes **(**Fig. 2J**)**, CD115^+^ monocytes **(**Fig. 2K**)**, Ly6G^+^ neutrophils **(**Fig. 2L**)** at day 5 of differentiation, a phenotype reflecting the changes in BM composition after rivaroxaban treatment in vivo (see Fig. 1).

**Fig. 3:**
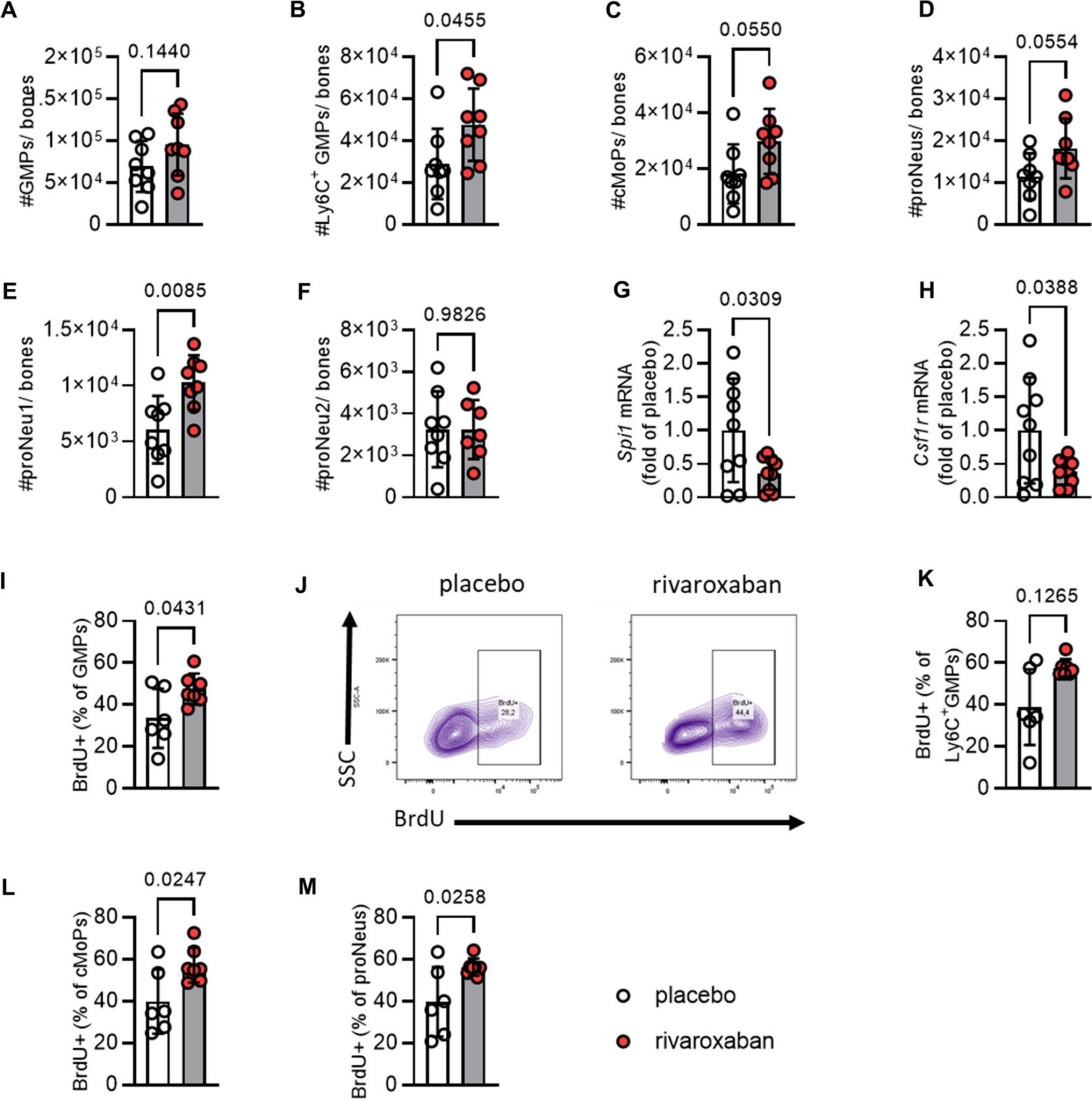
Rivaroxaban decreases specific GMP subsets in the bone marrow at steady state. C57BL6/J mice were treated with placebo or rivaroxaban for 7 days. Flow cytometric analysis of the bone marrow from tibiae and femora. **A** Total granulocyte–monocyte progenitors (GMPs; Lin^−^Sca1^−^c-Kit^+^CD34^+^CD16/32^+^), **B** Ly6C^+^ GMPs (Lin^−^Sca1^−^c-Kit^+^CD34^+^CD16/32^+^Ly6C^+^), **C** common monocyte progenitors (cMoPs; Lin^−^Sca1^−^c-Kit^+^CD34^+^CD16/32^+^Ly6C^+^CD135^−^CD115^+^), **D** proNeutrophiles (proNeus; Lin^−^Sca1^−^c-Kit^+^CD34^+^CD16/32^+^Ly6C^+^CD135^−^CD115^−^),** E** proNeus1 (Lin^−^Sca1^−^c-Kit^+^CD34^+^CD16/32^+^Ly6C^+^CD135^−^CD115^−^CD11b^low^CD106^−^), and **F** proNeus2 (Lin^−^Sca1^−^c-Kit^+^CD34^+^CD16/32^+^Ly6C^+^CD135^−^CD115^−^CD11b^high^CD106^+^) shown as absolute cell numbers (#); *n* = 8. mRNA expression of **G** spleen focus forming virus (SFFV) proviral integration oncogene (*Spi1*) and **H** colony-stimulating factor 1 receptor (*Csf1r*) in isolated c-Kit^+^ bone marrow cells from rivaroxaban and placebo-treated mice. *n* = 9. C57BL/6 J mice were treated with either placebo or rivaroxaban for 7 days, followed by injection of bromodeoxyuridine (BrdU) 1 h before sample collection. The bone marrow cells from the tibiae and femora were then analyzed using flow cytometry to assess their proliferation. Quantification of BrdU^+^ **I**, GMPs and **J** the respective flow plot. Quantification of BrdU^+^, **K** Ly6C^+^ GMPs, **M** cMoPs, and **N** proNeus shown as percentages. *n* = 6–7. Data represent mean ± SD. *P < 0.05, statistical significance was determined by unpaired Student’s *t* test. SSC indicates side scatter

After observing alterations in progenitor cells during the differentiation process on day 3, we proceeded to examine the impact of rivaroxaban on crucial receptors and transcription factors that promote the differentiation of GMPs into monocytes and neutrophils [31, 39] (Fig. 3A). While the mRNA expression levels of kruppel-like factor 4 (*Klf4*, Supplementary Fig. 14B), CCAT enhancer binding proteins (*Cebp*a, Supplementary Fig. 14C) and *Cebpb* (Supplementary Fig. 14D), GM-CSF receptor (*Csfr2b,* Supplementary Fig. 14E), interferon regulatory factor 8 (*Irf8*, Supplementary Fig. 14F), and nuclear receptor 4A1 (*Nr4a1*, Supplementary Fig. 14G) were unaltered, the transcription factor spleen focus forming virus proviral integration oncogene (*Spi1,* also known as PU.1, Fig. 2G**)** and the colony-stimulating factor 1 receptor (*Csf1r*, Fig. 2H**)** encoding for the M-CSF receptor CD115 were significantly downregulated by rivaroxaban 72 h after differentiation.

### Rivaroxaban inhibits differentiation of neutrophil progenitor proNeu1 in vivo

Having obtained a first hint of rivaroxaban's effects in vitro, we tried to verify these findings also in the in vivo setting. While an increase in GMPs was detected in vitro, this effect was not directly mimicked in vivo and only a slight trend toward increased numbers was detected (Fig. 3A). However, recent studies underlined the importance of specific GMPs subsets with heterogeneous transcriptomic and proteomic signatures [43]. We therefore investigated the effects of rivaroxaban on these GMP subsets in vivo and performed extensive flow cytometric analyses. While a minor rise in the population of Ly6G^+^ GMPs (Fig. 3B) was detected, common monocyte progenitors (cMoPs, Fig. 3C) and a distinct neutrophil progenitor subset, termed as proNeus (Fig. 3D), were significantly increased in animals treated with rivaroxaban. Intriguingly, we discovered significant effects on the slightly more differentiated GMP subsets, specifically proNeus1 (Fig. 3E). In contrast, no effect was observed in the more differentiated proNeus2 (Fig. 3F) after rivaroxaban treatment.

Furthermore, we aimed to examine whether the downregulation of the transcription factors *Spi1* and *Csf1r*, which was observed in vitro, could also be detected in vivo. To specifically analyze the respective expression in HSPCs, we isolated c-kit positive cells and conducted qPCR analyses. Indeed, the results confirmed the in vitro data and revealed a significant downregulation in the expression of both transcription factors in mice treated with rivaroxaban (Fig. 3 G–H).

In addition, we assessed BrdU labeling to determine the proliferation rate of each progenitor subset in vivo, since not only a downregulation of transcription factors relevant for cell maturation, but also cell proliferation might be affected. Notably, there was an overall elevation in the number of BrdU-positive GMPs (Fig. 3I), primarily observed in the proNeus (Fig. 3M) and cMoPs (Fig. 3L) subsets. Conversely, no significant alterations were noted in the Ly6C^+^ GMPs (Fig. 3K).

### Cellular mechanisms underlying differentiation arrest by rivaroxaban

**Fig. 4:**
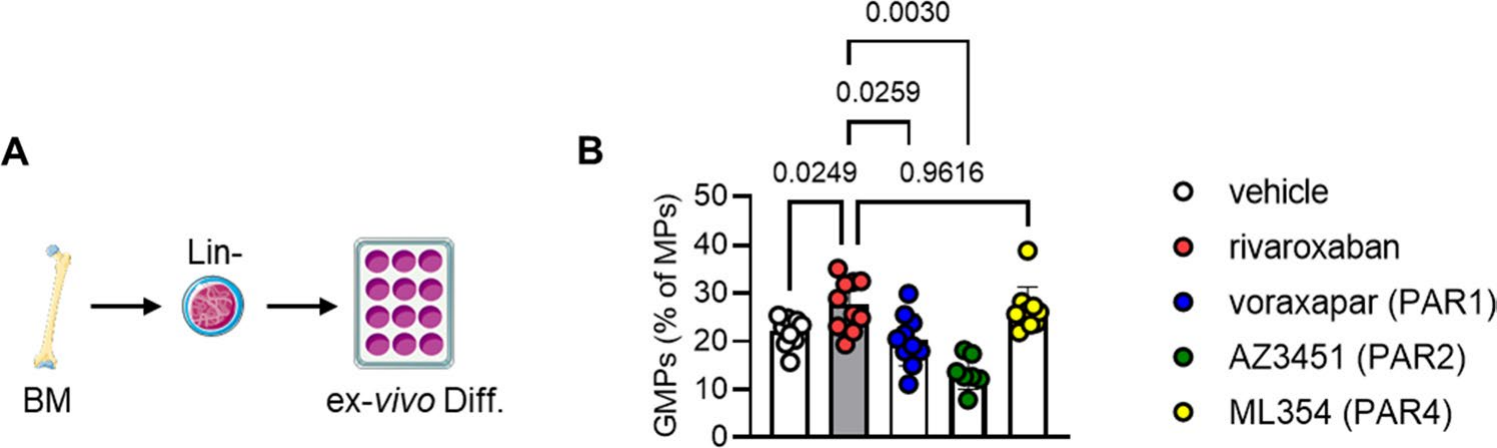
PAR antagonists do not completely mimic rivaroxaban`s effects on GMP. Ex vivo differentiation of bone marrow derived (Lin)eage- cells into myeloid progenitors (MPs) (d3). Cells were treated with 10 μM rivaroxaban, voraxapar (PAR1 antagonist), AZ3451 (PAR2 antagonist), 200 nM ML354 (PAR4 antagonist), or the respective vehicle control and analyzed by flow cytometry. **A** Experimental design. Quantification of **B** granulocyte–monocyte progenitors (GMPs; Lin^−^Sca1^−^c-Kit^+^CD34^+^CD16/32^+^), *n* = 6–9. Data represent mean ± SD. *P < 0.05, Statistical significance was determined by mixed-effects analysis, followed by Sidak post hoc test

The observed effects of rivaroxaban in BM-derived cells suggested the involvement of signaling through FXa-responsive PAR receptors. Thus, we evaluated differentiation in the presence of rivaroxaban or specific antagonists against PAR1 (vorapaxar**)**, PAR2 (AZ3451**)** and PAR4 (ML354) (Fig. 4B). Since the amount of FXa in the cell culture was very low (3.7 ng/ml as determined by an enzyme-linked immunosorbent assay (ELISA, data not shown)) and not all PAR receptors exhibit the same affinity to FXa, we decided to supplement FXa in these experiments.

The PAR2 agonist did not mimic the effects of rivaroxaban on myeloid progenitors GMPs, and in contrast rather exhibited opposite effects to those detected with rivaroxaban, similar to the PAR1 antagonist. However, antagonizing PAR4 by trend mimicked rivaroxaban's effect on GMP differentiation (vehicle: 22.19 ± 2.83; rivaroxaban: 27.59 ± 5.03 versus PAR4 antagonist ML354: 26.66 ± 4.45 GMPs (% of MPs)), thereby suggesting at least a partial contribution in mediating rivaroxaban's effects.

### No alterations in apoptosis, splenic release, and homing of circulating immune cells by rivaroxaban

While treatment with the FXa inhibitor rivaroxaban was accompanied by decreased myeloid cell numbers in the BM (Fig. 1) primarily driven by effects on the differentiation and maturation of the specific GMP subset proNeu1 (Fig. 3), it did not affect the number of circulating leukocytes under homeostatic conditions (Supplementary Fig. 15, A–D). Based on this, we explored potential compensatory mechanisms. First, we examined whether apoptosis of circulating immune cells was affected; however, flow cytometric analysis using Annexin V staining did not reveal changes in apoptosis of circulating myeloid leukocytes (Supplementary Fig. 16).

Considering the spleen's role as an important secondary lymphoid organ also critically involved in immune responses, we verified whether it might mediate compensatory mechanisms. However, flow cytometric analysis of myeloid leukocytes, neutrophils, monocytes, and the respective monocyte subsets in the spleen did not show any changes upon treatment with rivaroxaban for 7 days (Supplementary Fig. 17).

Next, we investigated whether rivaroxaban affects immune cell trafficking and their ability to home to the hematopoietic niche. To this end, analysis of mRNA expression of important migration and retention factors, such as *Cxcl12*, *Cxcl2*, *Cxcl1*, and *Ccl3*, was performed in cells from the BM. While *Cxcl1* was not detectable under steady state conditions, no alterations in mRNA expression were found for *Cxcl12* and *Cxcl2* (Supplementary Fig. 18. A–B). However, there was a strong trend toward downregulation of *Ccl3* under basal conditions (Supplementary Fig. 18 C).

### Attenuated cardiac and systemic inflammation post-I/R in rivaroxaban-treated mice

**Fig. 5:**
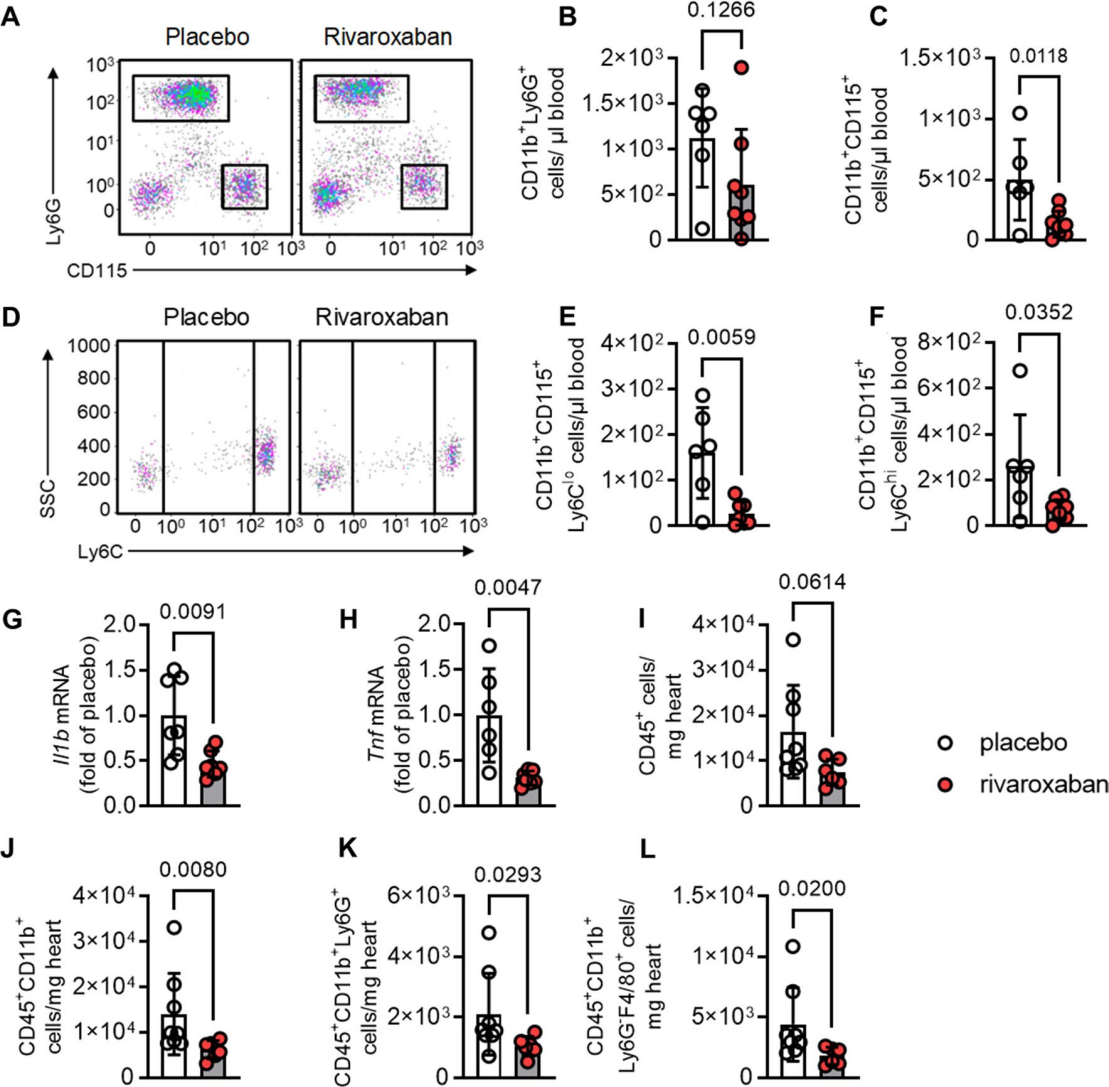
Attenuated cardiac and systemic inflammation in rivaroxaban-treated mice after myocardial infarction. C57BL/6 J mice were treated with placebo or rivaroxaban and subjected to cardiac ischemia and 72 h of reperfusion (I/R). Flow cytometric analysis of blood showing **A** flow plots and quantification of circulating **B** neutrophils (CD11b^+^Ly6G^+^) and **C** monocytes (CD11b^+^CD115^+^). **D** Flow plots and quantification of circulating **E** Ly6C^lo^ monocytes (CD11b^+^CD115^+^Ly6C^lo^) and **F** Ly6C^hi^ (CD11b^+^CD115^+^Ly6C^hi^) monocytes; *n* = 6–8. **G** Interleukin 1 beta (*Il1β*) and **H** tumor necrosis factor alpha (*Tnfα*) mRNA expression in the left ventricles. Flow cytometric analysis of cardiac tissue **(I–L)** and quantification of **I** cardiac leukocytes (CD45^+^), **J** myeloid leukocytes (CD45^+^CD11b^+^), **K** neutrophils (CD45^+^CD11b^+^Ly6G^+^), and **L** macrophages (CD45^+^CD11b^+^Ly6G^−^F4/80^+^). Data represent mean ± SD, *P < 0.05, **P < *0.01*. Statistical significance was determined by unpaired *t* test in **A**–**H** and Mann–Whitney test in **I**–**L**. SSC indicates side scatter. # indicates absolute cell numbers

After resolving rivaroxaban's effects under steady-state conditions, we wondered whether they persist under strongly activated emergency hematopoiesis as found in the acute phase of cardiac I/R. This situation is characterized by reduced immune cell retention in the BM and increased myeloid cell efflux into the circulation; thus, alterations in BM composition could become evident in both, the blood and the heart. To address this issue, mice were pretreated with rivaroxaban or vehicle for 7 days and subsequently subjected to cardiac I/R injury. As assessed by flow cytometry, rivaroxaban-treated mice exhibited substantially reduced levels of circulating neutrophils **(**Fig. 5A–B**)**, monocytes (Fig. 5A, C), and monocyte subsets **(**Fig. 5D–F**),** indicating attenuated systemic inflammation in response to FXa inhibition 72 h post-I/R. At the same time, this was accompanied by a diminished cardiac inflammation as indicated by decreased mRNA expression of interleukin-1 beta (*Il1b*, Fig. 5G**)** and tumor necrosis factor alpha (*Tnfα,* Fig. 5H**)** in left ventricles of rivaroxaban-treated mice. In line, flow cytometric analysis of cardiac immune cell composition revealed decreased leukocyte infiltration in rivaroxaban-treated mice **(**Fig. 5I**)**, as reflected by substantially reduced CD11b^+^ myeloid leukocyte **(**Fig. 5J**)** as well as neutrophil **(**Fig. 5K**)** and macrophage **(**Fig. 5L**)** numbers. Of note, under both homeostatic conditions and I/R, the counts of circulating lymphocytes remained unaffected (Supplementary Fig. 19). Importantly, solely rivaroxaban (without subsequent I/R) did not affect at all cardiac immune cell composition (Supplementary Fig. 20). Infarct size and cardiac function were evaluated using TTC staining, echocardiography, and MRI. The results indicated that treatment with rivaroxaban had no impact on functional parameters, such as ejection fraction, end-systolic volume, end-diastolic volume, or the size and area at risk of the infarct (Supplementary Fig. 21).

### Rivaroxaban attenuates the early neutrophil response post-I/R

We next addressed the puzzling question of the underlying reason for the reduced immune cell numbers in the blood and infarcted myocardium in rivaroxaban-treated animals 72 h post-MI. As seen in homeostatic conditions, rivaroxaban did not affect HSPCs as well as lymphocyte numbers in response to I/R (Supplementary Figs. 10, 15E–H, 19).

**Fig. 6:**
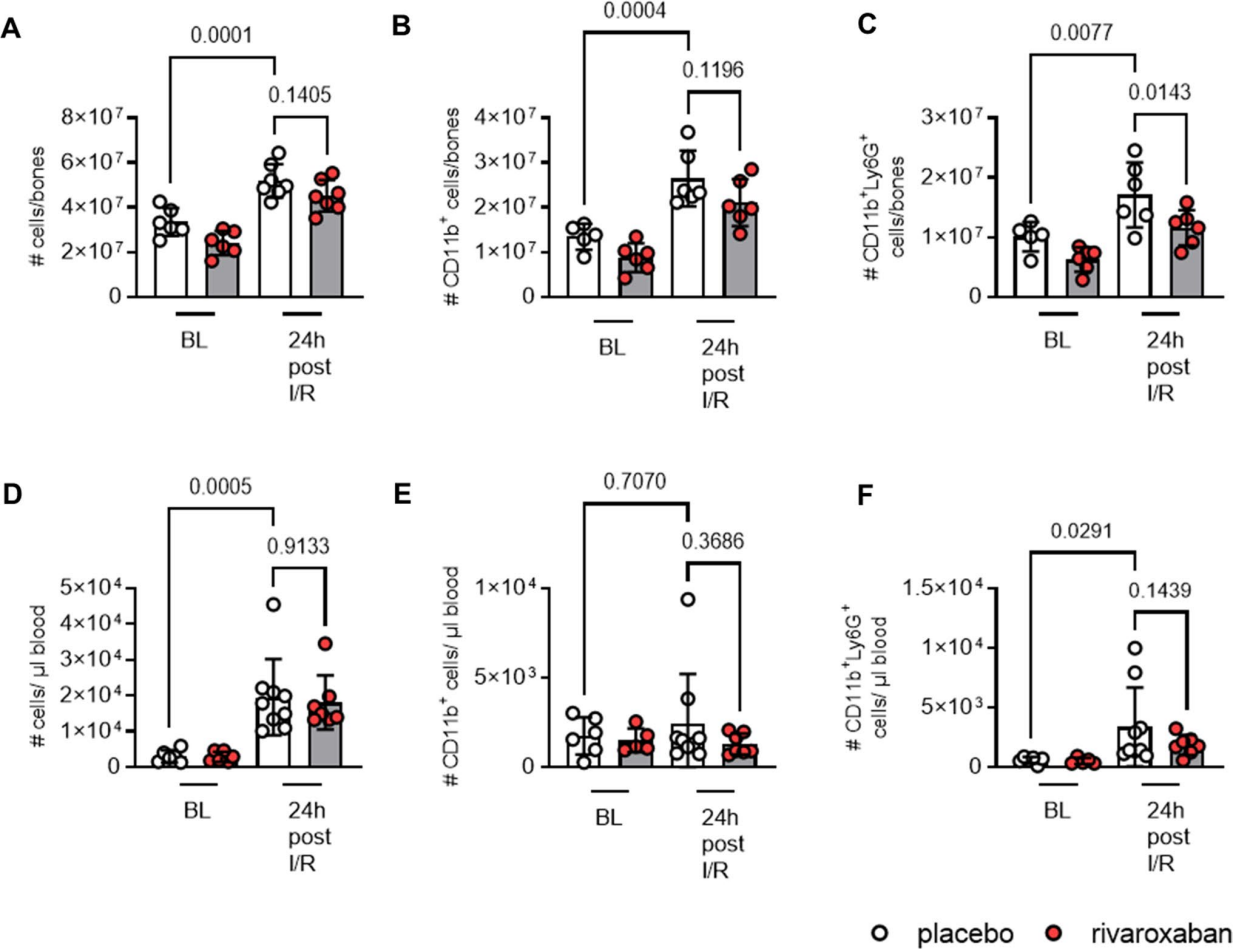
Rivaroxaban attenuates the early neutrophil response post-I/R. Flow cytometric analysis of the bone marrow (BM) from tibiae and femora and cardiac blood after 7 days of feeding (BL) and 24 h post-cardiac ischemia and reperfusion (I/R) showing **A** all cells, **B** myeloid leukocytes (CD11b^+^), **C** neutrophils (CD11b^+^Ly6G^+^) in the BM, and **D** all cells. **E** Myeloid leukocytes (CD11b^+^), **F** neutrophils (CD11b^+^Ly6G^+^) in the circulation. *n* = 5–10. Data represent mean ± SD; *P < 0.05, **P < 0.01, ***P < 0.001. Statistical significance was determined by ordinary one-way ANOVA, followed by Sidak post hoc test

Here, we focused in particular on neutrophils, due to the specific effects of rivaroxaban for the GMP subset proNeu1 observed under homeostatic conditions (see above), and—since neutrophils are known to be recruited rapidly post-I/R—on an early time point, i.e., 24 h post-IR. Indeed, we observed significantly decreased numbers of mature neutrophils in the bone marrow at baseline and also 24 h post-I/R in rivaroxaban-treated mice (Fig. 6). While neutrophils were found to be significantly decreased in the bone marrow, only a trend toward a reduction was observed in the blood 24 h after I/R under rivaroxaban treatment (Fig. 6). Accordingly, mRNA expression levels of *Spi1* and *Csf1r* were also not different between both treatment group (data not shown). Thus, this time point (24 h post-I/R) might have already been too late to hit the peak of the first wave of neutrophils being released (with significant differences between the groups). Nevertheless, since the acute neutrophil response is critically determining the second wave of inflammation and also decisive for the reparative phase post-MI, it is most likely that the observed impairment of neutrophil maturation under rivaroxaban pretreatment leads to the attenuation of the subsequent immune cell release into the blood und recruitment to the injured heart.

### Circulating leukocytes in STEMI patients treated with rivaroxaban

Based on our findings of reduced numbers of circulating leukocytes in mice post-I/R **(**Fig. 5A-F), we finally aimed to elucidate whether similar findings might translate into ST-elevation myocardial infarction (STEMI) patients after primary percutaneous coronary intervention (pPCI).

**Fig. 7:**
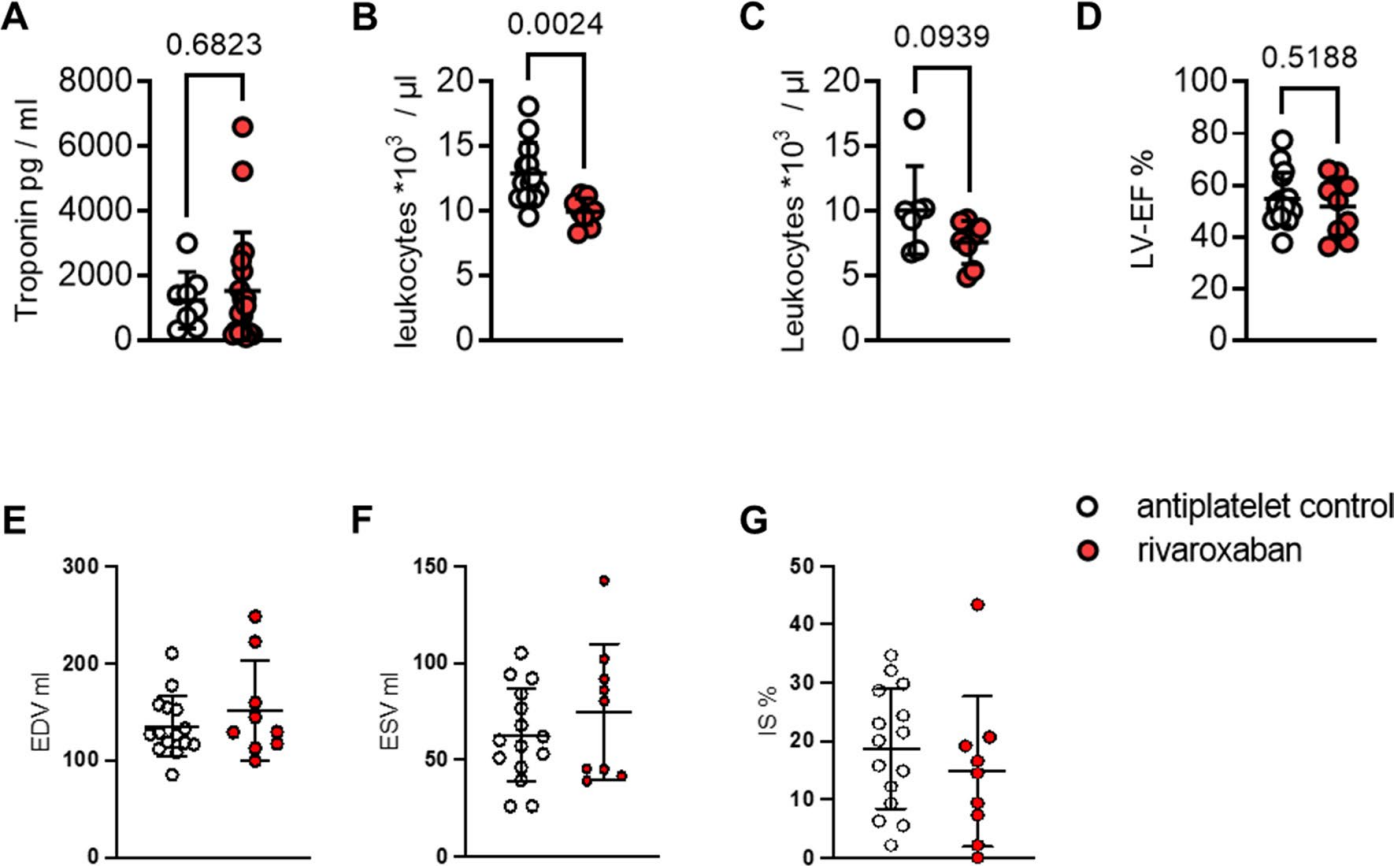
Reduced circulating leukocytes and unaltered cardiac function in STEMI patients treated with or without rivaroxaban. Patients received either antiplatelet therapy alone or in addition to rivaroxaban. Blood sample collection was performed at the day of cardiac catheterization (d0) and 4 days later (d4). A hemocytometer was used to determine cell counts. **A **Troponin levels (d0) displayed in pg/ml (antiplatelet control: n = 18, 8; rivaroxaban: *n* = 8). **B **Leukocytes (d0) and **C** leukocytes (d4) shown as 10^3^ cells/µl blood (antiplatelet control: *n* = 13, 8; rivaroxaban: *n* = 9, 7). To assess cardiac function, magnetic resonance imaging was performed between day 4 and 6. **D** Left ventricular ejection fraction (LV-EF); **E** end-diastolic volume (EDV) in ml. **F** End-systolic volume (ESV) in ml. **G**, infarct size (IS) as % of total myocardial mass (antiplatelet control: *n* = 15; rivaroxaban: *n* = 9). Data represent mean ± SD; *P < 0.05, **P < 0.01. Statistical significance was determined by unpaired *t* test in **A–B** and Mann–Whitney test in **C–F**

Therefore, we analyzed hemograms from STEMI patients receiving antiplatelet medicine or in combination with rivaroxaban (Fig. 7). Baseline characteristics and medication of patients are displayed in Supplementary Table 6; troponin levels were similar between both treatment groups (Fig. 7A).

Similar to our findings in mice, circulating leukocyte numbers were markedly reduced in the rivaroxaban group as compared to the control group during the acute post-ischemic phase, **(**Fig. 7B**)**, but by day 4 this difference did not reach anymore the level of statistical significance. (Fig. 7C). Interestingly, also similar to our murine findings, cardiac function as determined by MRI was not altered between the control and the rivaroxaban group (Fig. 7D–G).

## Discussion

This study reports a yet unknown effect of rivaroxaban in controlling GMP differentiation and myelopoiesis in the BM, with major consequences for immune cell recruitment after myocardial infarction. Our data suggest that FXa inhibition by rivaroxaban skews BM-derived HSPCs toward myeloid lineages and blocks their further differentiation at the level of a specific GMP subset, proNeu1, which has recently been reported as the final branching point for neutrophils and neutrophil-like monocytes under inflammatory conditions [31]. As underlying cause, we mechanistically found a downregulation of *PU.1* and *Csf1r,* two major regulatory transcription factors in GMP to neutrophil/monocyte transition, in isolated progenitor cells [25, 39, 53, 70]. Importantly, it has been reported that proliferation of intermediate myeloid progenitors is essential before differentiation takes place [57]. The delicate balance between proliferation and differentiation is tightly controlled and disruption of expression of these transcription factors can impede the induction of further differentiation, resulting in an extended proliferation phase [57] as also observed in our study.

Of note, under homeostatic conditions and without any inflammatory trigger, white blood cell counts were unaffected by rivaroxaban treatment. Here, counteracting mechanisms which might compensate for the reduction in myelopoiesis could not be identified. Similarly, splenic leukocytes as well as apoptosis of circulating leukocytes were not altered. Also, migration and retention factors in the bone marrow such as *Cxcl12* (also known as *Sdf1a*), *Cxcl1*, *Cxcl2*, and *Ccl3* (also known as MIP-1α) were not changed between treatment groups; only a trend toward downregulation of *Ccl3* was detected. *Ccl3* has been described as an inflammatory cytokine [6], its downregulation may also contribute to the anti-inflammatory effect of rivaroxaban. Further, *Ccl3* has been reported as a suppressor of HSC proliferation [5, 13], which aligns with our findings of increased proliferation in myeloid progenitor cells. Notably, Staversky et al*.* [65] reported that mice lacking *Ccl3* exhibited reduced populations of mature myeloid cells but an increase in myeloid progenitors and HSPCs. Therefore, it seems most likely that the steady state demand of circulating leukocytes can be maintained while the inhibition of cell maturation only becomes evident in situations of elevated leukocyte efflux from the bone marrow to the blood as seen in cardiac I/R.

Importantly, the ability of rivaroxaban to suppress BM myelopoiesis by leukocyte differentiation arrest appears to be restricted to homeostatic conditions and abolished during emergency hematopoiesis. Indeed, reduced mRNA expression of the respective transcription factors under rivaroxaban treatment was limited to steady state conditions and no longer observable post-I/R (data not shown). Our finding that rivaroxaban-treated STEMI patients also exhibited reduced numbers of circulating leukocytes as compared to antiplatelet therapy alone, provide a hitherto unsuspected impact of rivaroxaban on hematopoietic leukocyte maturation in the BM. Accordingly, in response to cardiac I/R, alterations in BM composition were likely reflected by changes in circulating blood cells, especially at early time points driven by neutrophil efflux from the bone marrow. Nevertheless, since the acute neutrophil response is critically determining the second wave of inflammation and also decisive for the reparative phase post-MI [18, 55], it is most likely that the observed impairment of neutrophil maturation under rivaroxaban pretreatment leads to the attenuation of the subsequent immune cell release into the blood und recruitment to the injured heart. Indeed, at 72 h post-I/R fewer circulating and cardiac monocytes were observed despite unaffected myelopoiesis at that particular time point.

Extravascular coagulation within the BM niche has been attributed a key role in the regulation of HSPC maintenance and activation (reviewed in [67]). Since FX/FXa was shown to be actively produced by osteoblasts, osteoclasts, and megakaryocytes within the extravascular BM niche [58, 66] and prothrombin (also known as FII), a target of FXa, has been detected in the bone matrix of tibia and femur [34], a specific role of FXa in extravascular coagulation was suggested. However, the role of FXa within the BM niche and the consequences of pharmacological FXa inhibition on hematopoiesis at steady state and under stress conditions are only poorly understood.

Evidence exists that low thrombin levels in the BM microenvironment at steady state promote LT-HSC retention through activation of anticoagulant protein C (aPC)–endothelial protein C receptor (EPCR)-PAR-1 signaling [26, 60]. Considering the crucial role of FXa in thrombin generation, we hypothesized that pharmacological inhibition of FXa impairs HSC retention through decreased thrombin signaling. However, rivaroxaban treatment neither altered the number of HSPCs in general nor that of LT-HSCs in particularly**.** In contrast, we found decreased myeloid leukocyte numbers in the BM of rivaroxaban-treated mice indicating an important role for FXa in myeloid cell survival and/or maturation. Recently, inhibition of FXA-PAR-2 signaling by rivaroxaban was shown to promote macrophage autophagy [52], a process which can either prevent or promote cell death, e.g., through differential regulation of apoptosis (reviewed in [42]). Furthermore, autophagy has been implicated in both, the survival and differentiation of monocytes [69]. While myeloid cell apoptosis was unaffected*,* myeloid cell differentiation was blocked by rivaroxaban.

Given that the cellular actions of FXa are critically dependent on the activation of PARs, we hypothesized that rivaroxaban-induced modification of myeloid progenitor differentiation was PAR dependent and could be mimicked by PAR inhibition. PAR-1 is a negative regulator of hematopoietic differentiation in murine embryonic stem cells [69] and HSPCs were shown to express the EPC receptor (EPCR), a target for FXa and co-receptor of PAR-1 [68]. While aPC-dependent EPCR-PAR-1 signaling promotes HSC retention as mentioned before, high thrombin levels were shown to facilitate HSC mobilization through direct proteolytic cleavage of PAR-1. Finally, loss of EPCR function on both, BM stromal and hematopoietic cells, gives rise to BM failure [49] in mice. PAR-2 was shown to promote osteoblast and osteoclast differentiation [20] and PAR-2 in myeloid cells was identified as a crucial regulator of fetal liver erythropoiesis [59], but does not affect steady-state erythropoiesis in the adult BM. Although PAR-4 was shown to be expressed in the BM of long bones, but not in BM-derived stem cells [63], the role of PAR-4 in myeloid progenitor differentiation is yet incompletely understood. However, none of the PAR antagonists mimicked the effects of rivaroxaban on myeloid progenitor differentiation completely. It has been reported that PAR1 and PAR4 can form heterodimers [4, 40], and on human platelets, PAR1 acts as a cofactor for PAR4, enhancing its cleavage [40]. Therefore, it seems that potentially PAR heterodimerization and subsequently altered functional cooperativity are needed to fully resemble rivaroxaban's effects. Surprisingly, another FXa inhibitor, apixaban, did not mimic rivaroxaban's effects in vitro. However, although both compounds are supposed to act as small molecule factor Xa inhibitors, differences in their kinetic functionality might be assumed to contribute to these outcomes [36].

Our finding of attenuated cardiac inflammation in rivaroxaban-treated mice is in line with a recent study investigating the effects of direct oral anticoagulants on inflammation in myocardial I/R injury [19]. Mechanistically, restricted cardiac inflammasome activation under control of anticoagulant protein C signaling was suggested. In consistency with these data, we found reduced cardiac *Il1b* and *Tnf* expression in the left ventricles of rivaroxaban-treated mice, accompanied by decreased neutrophil and macrophage infiltration after 72 h of I/R. Since rivaroxaban was also shown to attenuate leukocyte adhesion to the microvasculature [43], the currently proposed anti-inflammatory mechanisms include mainly alterations in the cardiac environment related to decreased leukocyte recruitment.

However, we were surprised to discover a profound reduction in circulating neutrophils and monocytes in response to FXa inhibition, indicating hitherto unknown actions of rivaroxaban beyond hemostasis. Our data suggest that reduced numbers of BM myeloid cells as a consequence of an attenuated maturation at steady state dampened the first wave of circulating neutrophils and monocytes post-I/R, leading to reduced cardiac leukocyte infiltration. Importantly, rivaroxaban did not compromise emergency myelopoiesis which is essential to ensure an adequate immune response toward a given pathogen or sterile insult [61]. However, it appears likely that due to attenuation the initial wave of neutrophil release and subsequent monocyte response an exaggerated cardiac inflammation might be prevented.

This is further supported by our preliminary data on reduced circulating leukocytes in rivaroxaban-treated STEMI patients. Notably, these effects were restricted to the acute phase post-MI but converged to control levels four days later. Furthermore, cardiac function in these patients was not altered compared to control. In sum, first results from patient samples resembled the findings of rivaroxaban treatment in mice, although they have to be further verified in larger cohorts.

In sum, our findings reported here not only confirm the previously reported anti-inflammatory properties of rivaroxaban, but also shed new light on its impact on remote organs like the bone marrow and its influence on myelopoiesis. This highlights the importance of considering the effects of rivaroxaban on myeloid cell production and function beyond its well-known anti-inflammatory actions.

### Supplementary Information

Below is the link to the electronic supplementary material.

Supplementary file1 (PDF 3665 KB)

## Data Availability

All data supporting the findings of this study are available within the article and its supplementary material.
