# Supplementary material for: Rivaroxaban attenuates neutrophil maturation in the bone marrow niche
Source: Basic Res Cardiol. 2023 Aug 14;118(1):31. doi: 10.1007/s00395-023-01001-5 (PMC10425524; doi:10.1007/s00395-023-01001-5)
Supplement: Supplementary file 1 — Supplementary file1 (PDF 3665 KB) [file 395_2023_1001_MOESM1_ESM.pdf]

Supplementary Figure S1

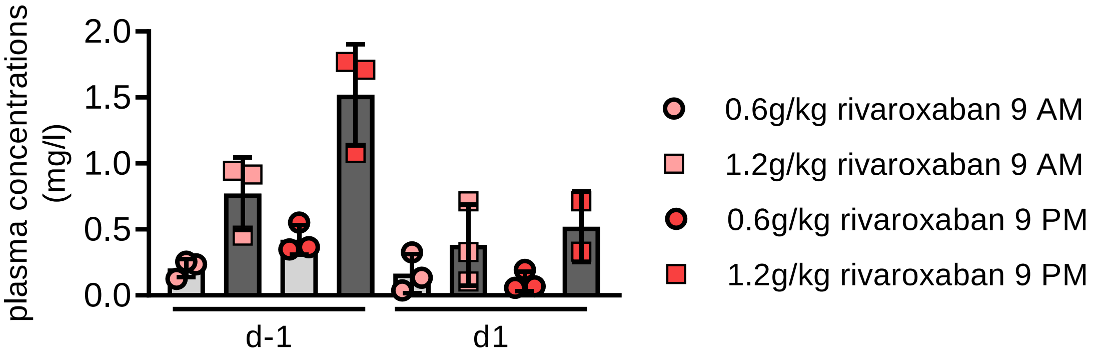

Figure S1. Rivaroxaban plasma levels. C57BL6/J mice were treated with 0.6 or 1.2g rivroxaban/kg diet for seven days prior to I/R injury. Rivaroxaban plasma concentration 24 hours prior to (d-1) or post (d1) cardiac I/R; n = 2-3. Cardiac blood was harvested at 9 AM or 9 PM. Data represent mean ± SEM.

Supplementary Figure S2

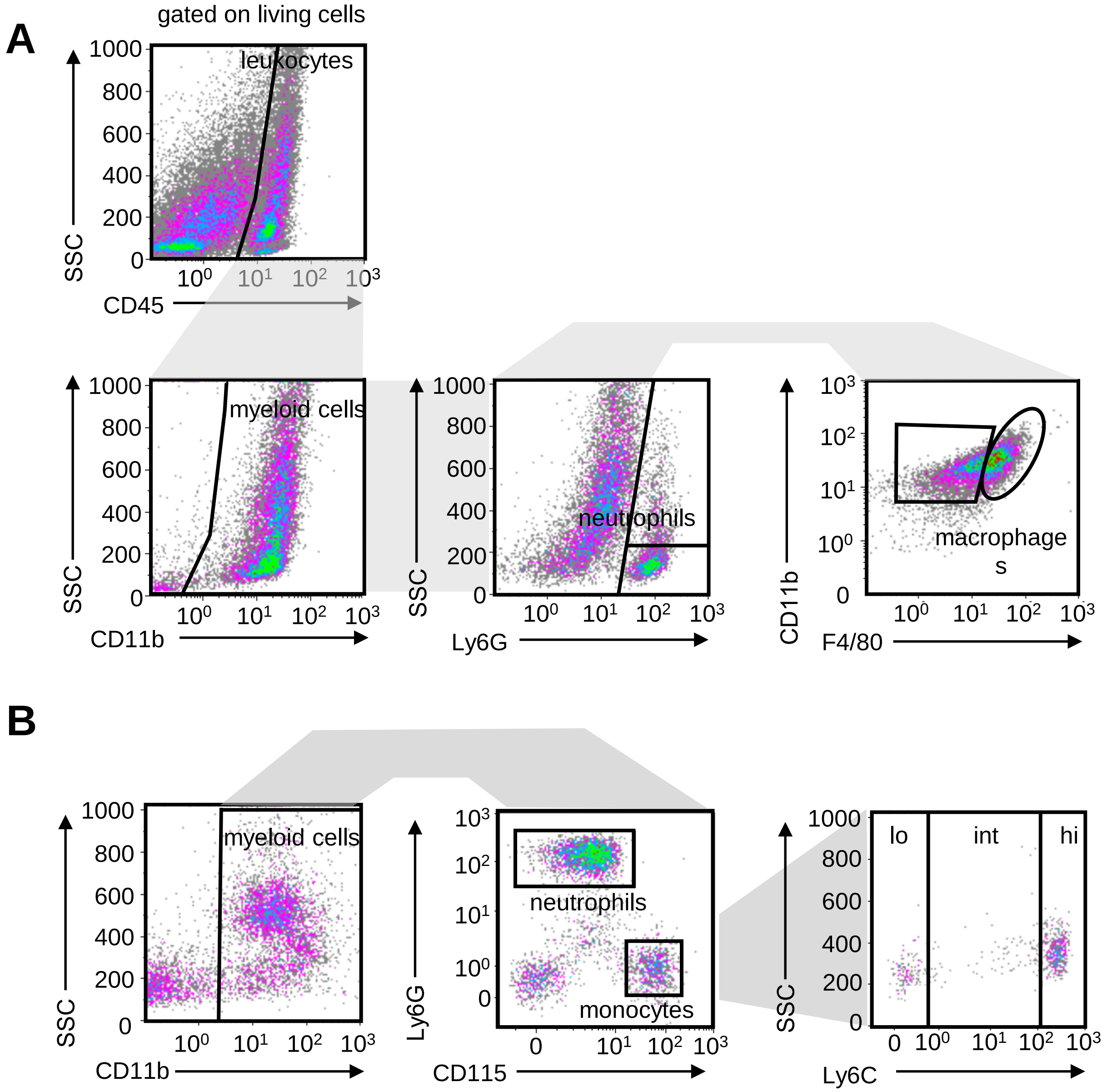

Figure S2. Gating scheme showing flow-cytometric analysis of cardiac tissue,spleen and blood. A, heart. **B**, blood and spleen.

Supplementary Figure S3

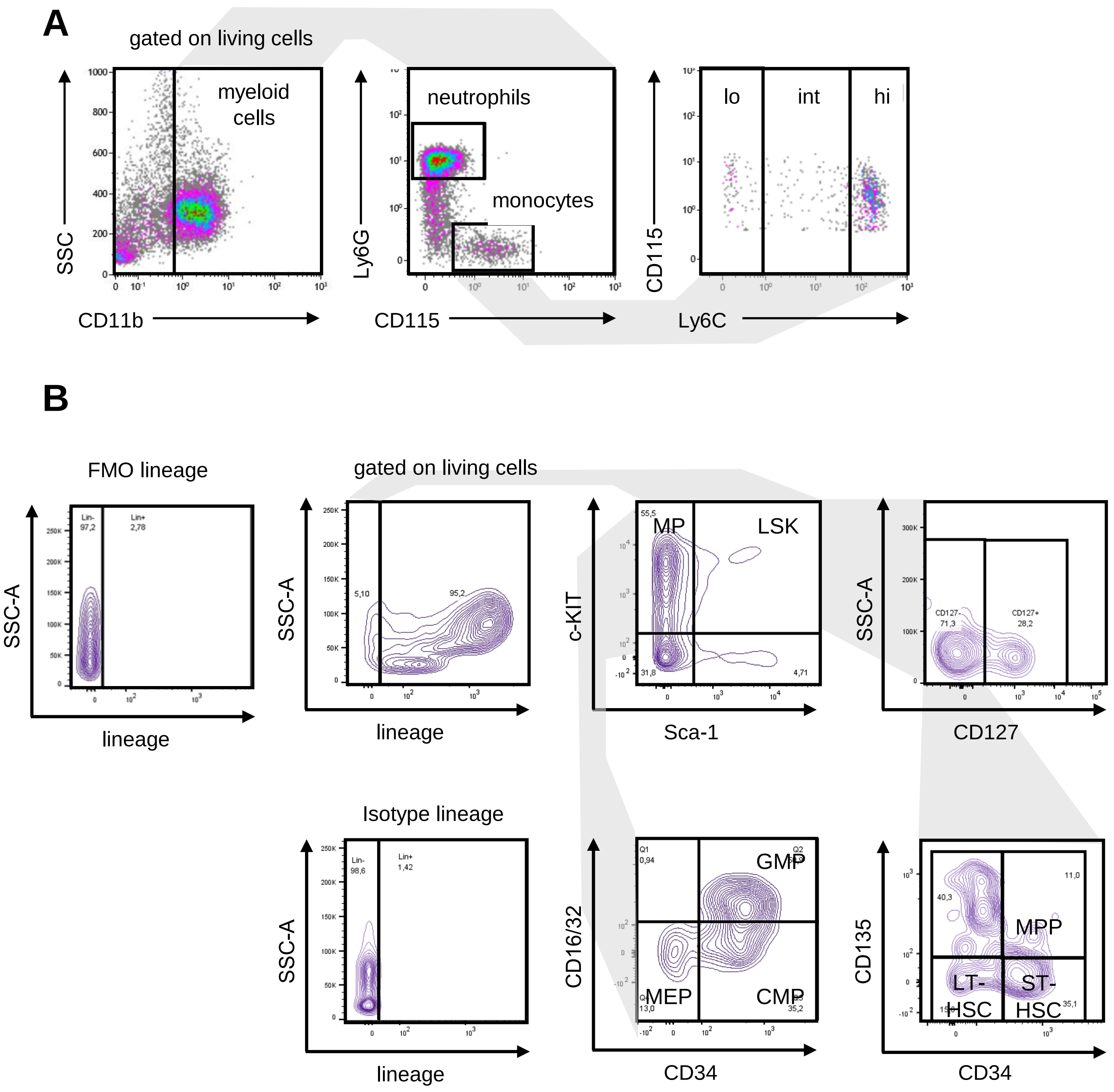

Figure S3. Gating scheme showing flow-cytometric analysis of bone marrow. A, bone marrow myeloid cells. **B,** bone marrow hematopoietic stem and progenitor cells (PMID 19023891). CMP = common myleoid progenitor cells; GMP = granulocyte-monocyte progenitor cells; LT-HSCs = long term hematopoietic stem cells; MEPs = megakaryocyte-erythrocyte progenitor cells; MP = myeloid progenitor cells; MPP = multipotent progenitor cells; ST-HSCs = short term hematopoietic stem cells.

Supplementary Figure S4

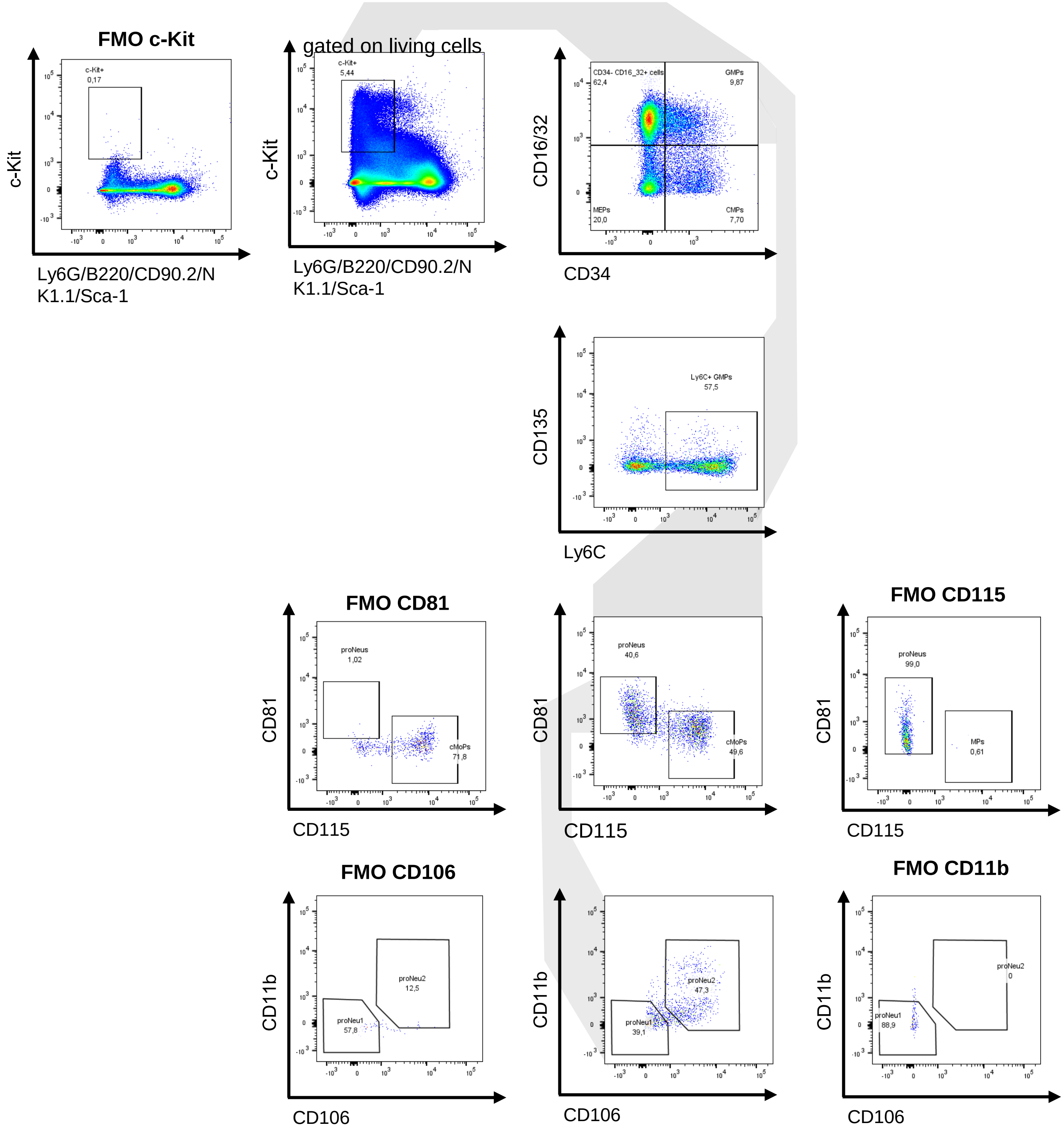

Figure S4. Gating scheme showing flow-cytometric analysis of bone marrow. CMP = common myleoid progenitor cells; GMP = granulocyte-monocyte progenitor cells; MEPs = megakaryocyte-erythrocyte progenitor cells; cMoP= common monocyte progenitor, proNeu = proNeutrophile. FMO indicates fluorescence minus one control

Supplementary Figure S5

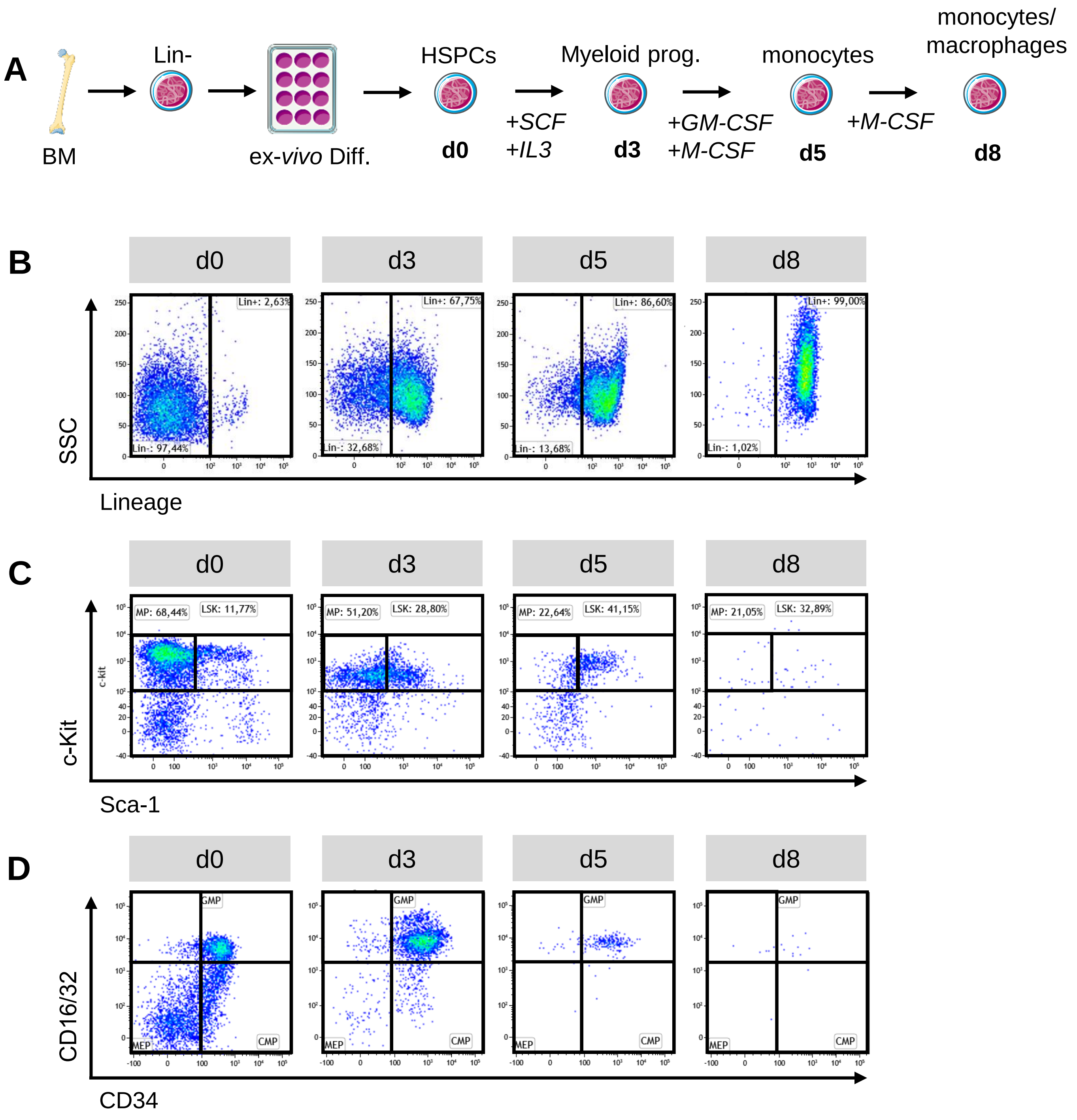

Figure S5. Loss of hematopoietic progenitor markers during *ex-vivo* differentiation into monocytes/macrophages. Bone marrow aspirates from wild-type (WT) mice were sorted via immunomagnetic bead separation. Lineage-depleted cells were differentiated *ex-vivo* into monocytes/macrophages. Differentiation stages were monitored by flow cytometry. **A,** experimental outline. Representative flow plots of **B,** lineage positive cells, **C,** myeloid progenitors (cKIT$^+$Sca1$^-$) and LSK cells (Lin$^-$cKIT$^+$Sca1$^+$), **D,** megakaryocyte-erythrocyte progenitors (MEPs; Lin$^-$cKIT$^+$Sca1$^-$CD34$^-$CD16/32$^-$), common myeloid progenitors (CMPs; Lin$^-$cKIT$^+$Sca1$^-$CD34$^+$CD16/32$^-$) and granulocyte-monocyte progenitors (GMPS; Lin$^-$cKIT$^+$Sca1$^-$CD34$^+$CD16/32$^+$) at day 0, 3, 5 and 8 of differentiation. Flow plots are gated on **B,** living cells, **C,** Lineage$^-$ cells, **D,** myeloid progenitors.

Supplementary Figure S6

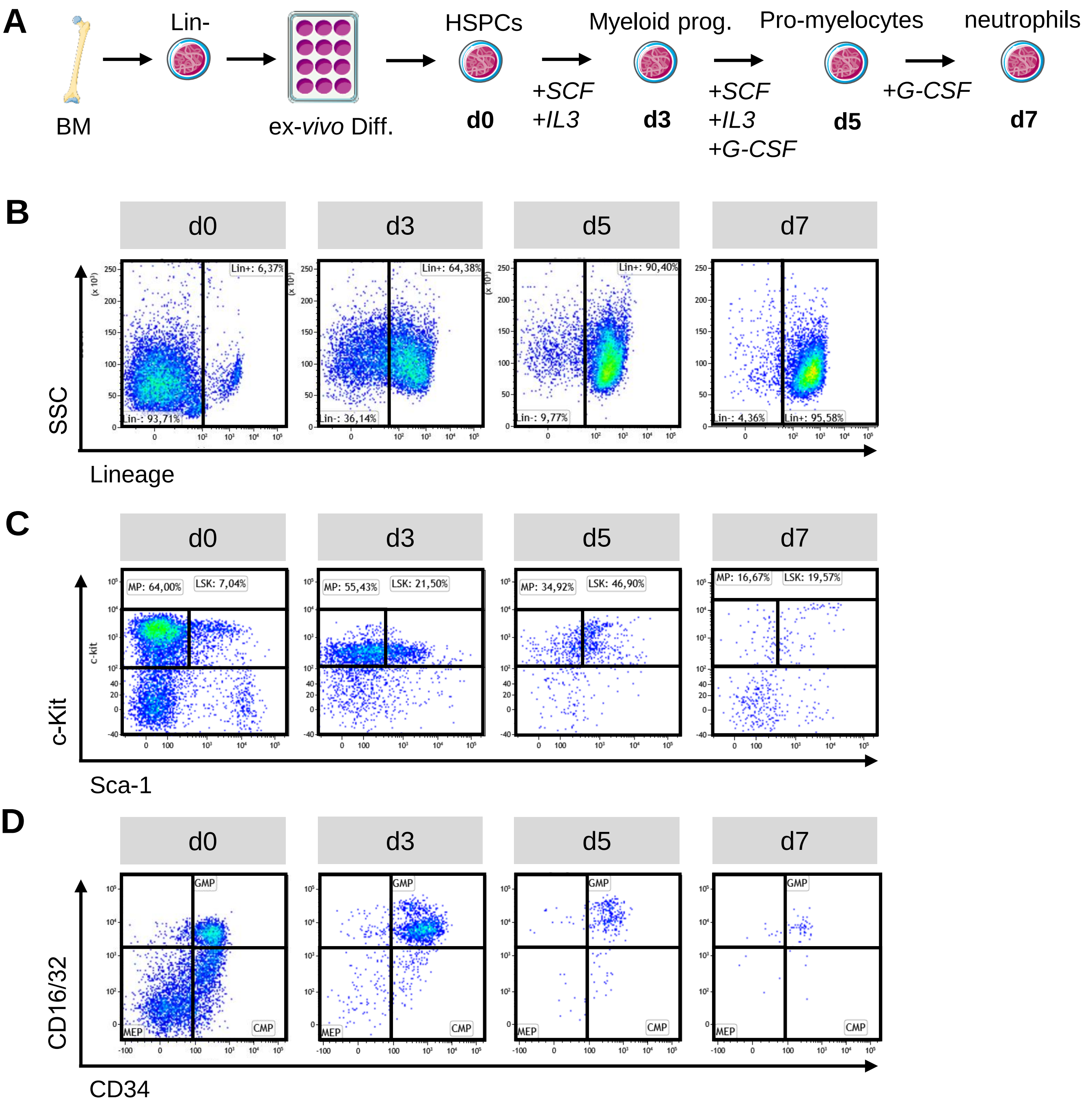

Figure S6. Loss of hematopoetic progenitor markers during *ex-vivo* differentiation into neutrophils. Bone marrow aspirates from wild-type (WT) mice were sorted via immunomagnetic bead separation using a Lineage cell depletion Kit. Lineage-depleted cells were differentiated *ex-vivo* into neutrophils. Differentiation stages were monitored by flow cytometry. **A,** experimental outline. Representative flow plots of **B,** lineage positive cells**, C,** myeloid progenitors (cKIT$^+$Sca1$^-$) and LSK cells (Lin$^-$cKIT$^+$Sca1$^+$), **C,** megakaryocyte-erythrocyte progenitors (MEPs; Lin$^-$cKIT$^+$Sca1$^-$CD34$^-$CD16/32$^-$), common myeloid progenitors (CMPs; Lin$^-$cKIT$^+$Sca1$^-$CD34$^+$CD16/32$^-$) and granulocyte-monocyte progenitors (GMPs; Lin$^-$cKIT$^+$Sca1$^-$CD34$^+$CD16/32$^+$) at day 0, 3, 5 and 7 of differentiation. Flow plots are gated on **A,** living cells, **B,** Lineage$^-$ cells, **D,** myeloid progenitors.

Supplementary Figure S7

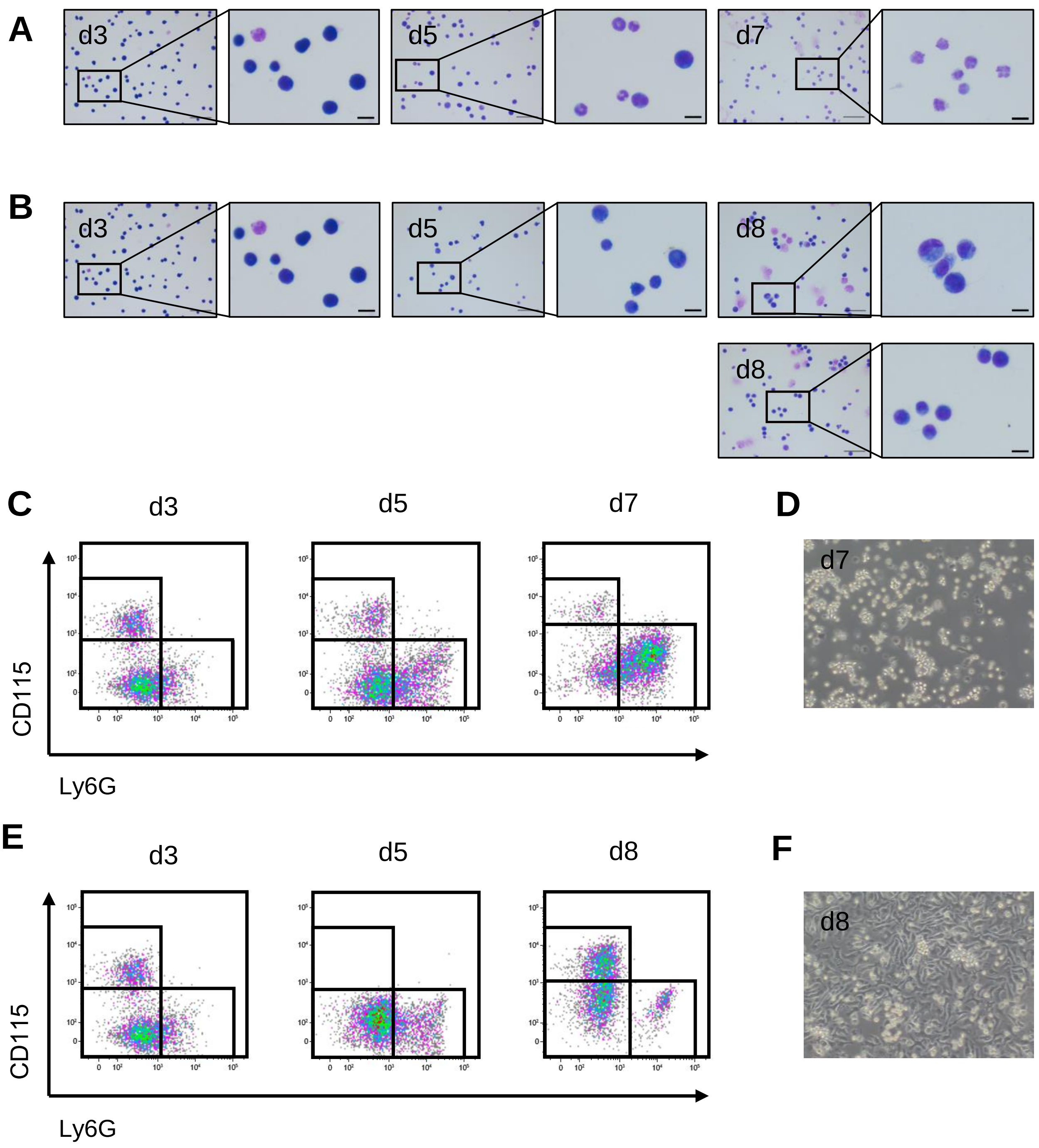

Figure S7. Differentiation of Lineage-depleted progenitor cells into myeloid leukocyte subsets. Bone marrow aspirates from wild-type (WT) mice were sorted via immunomagnetic bead separation. Lineage-depleted cells were differentiated *ex-vivo* into myeloid leukocytes. Representative Giemsa staining of cells at day 3, 5 and 7/8 during differentiation into **(A)** neutrophils and **(B)** monocytes/macrophages. Bars represent 50µm for overview images and 10µm for region of interests. Representative flow plots of **C,** neutrophil differentiation and **D,** respective microscopic picture of cells at day 7. Representative flow plots of **E,** monocyte/macrophage differentiation and **F,** respective microscopic picture of cells at day 8.

Supplementary Figure S8

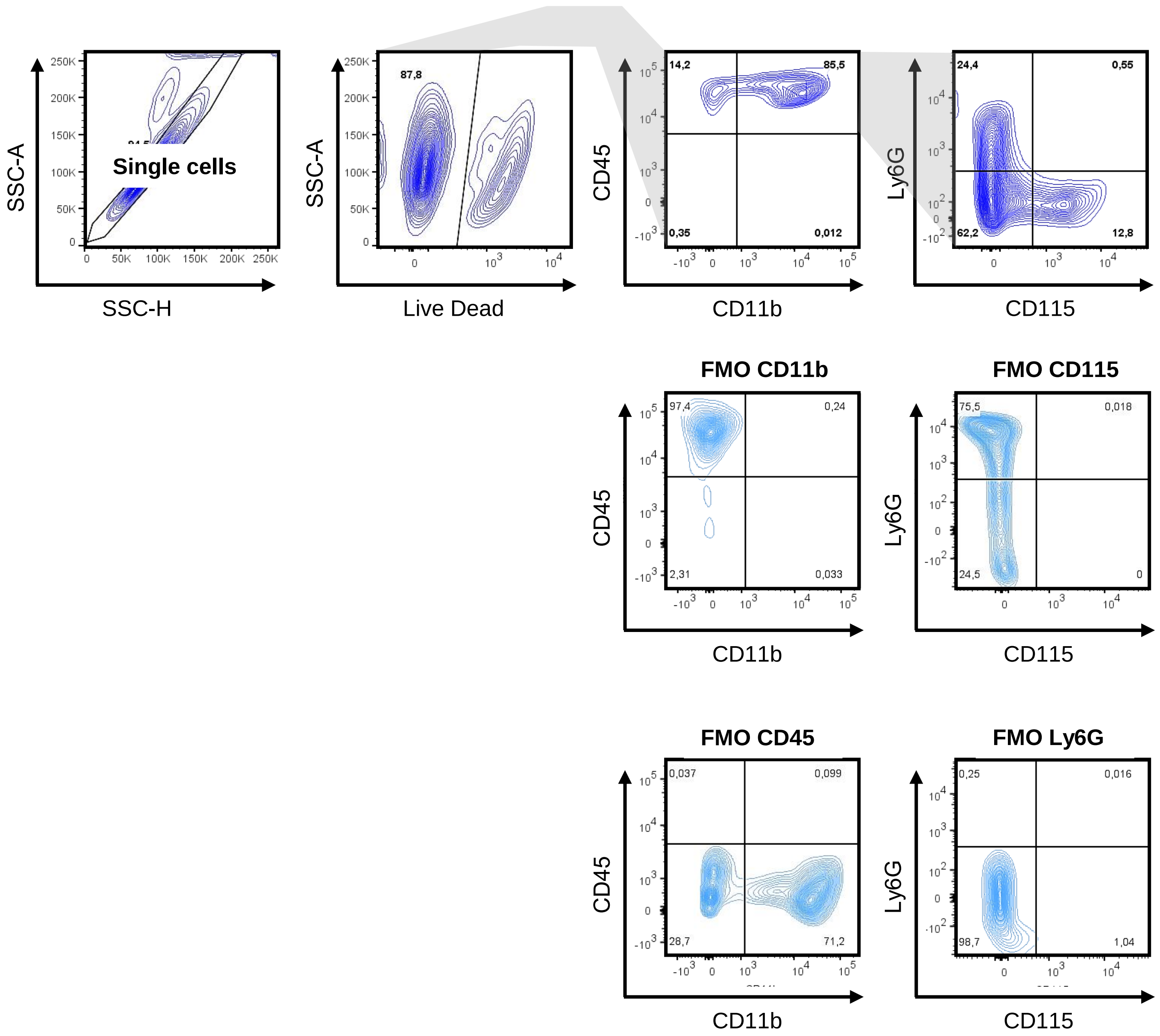

Figure S8. Gating scheme showing flow-cytometric anylsis of bone marrow derived leukocytes. FMO indicates fluorescence minus one control.

Supplementary Figure S9

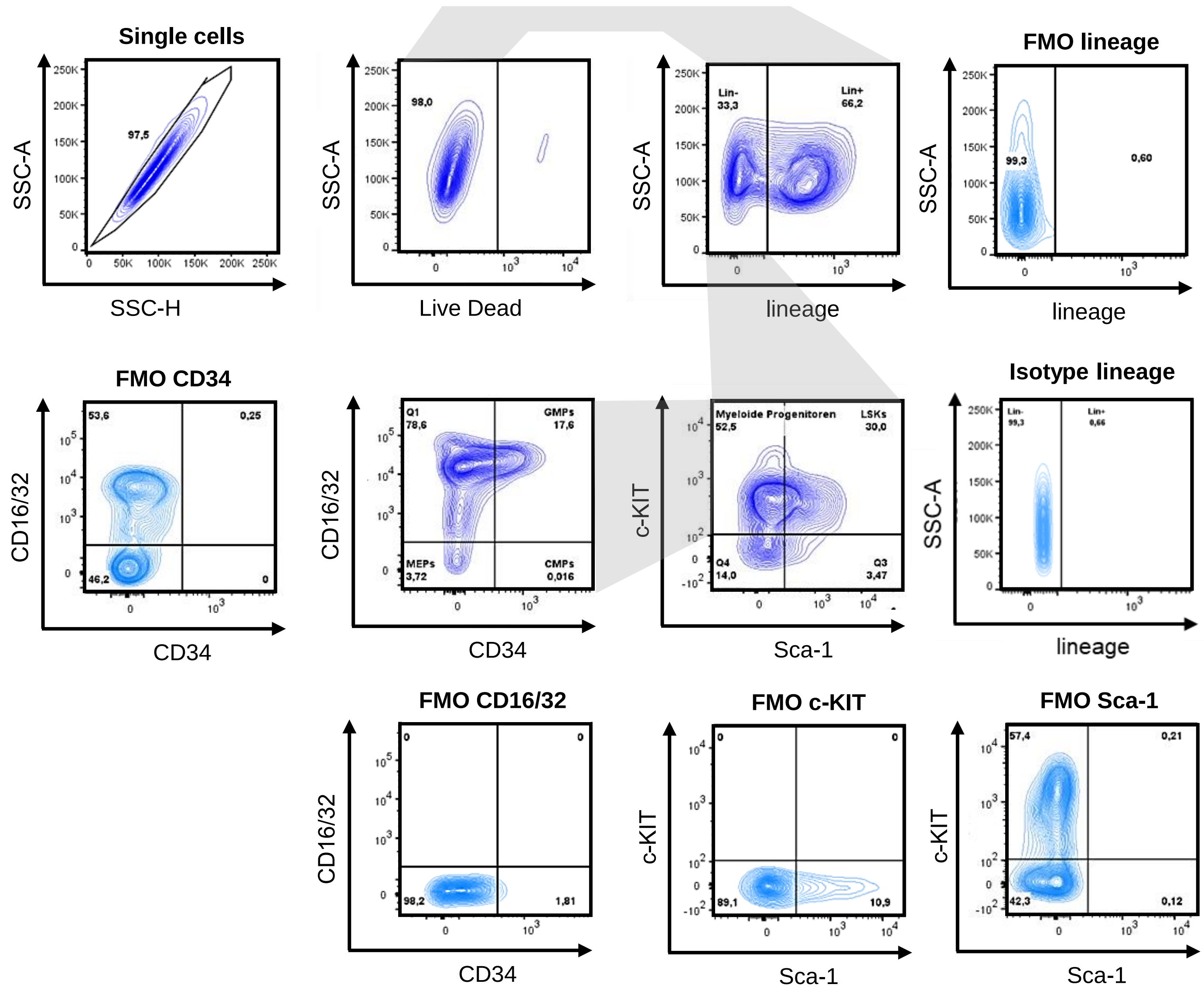

Figure S9. Gating scheme showing flow-cytometric analysis of bone marrow derived hematopoietic stem and progenitor cells. FMO indicates fluorescence minus one control.

Supplementary Figure S10

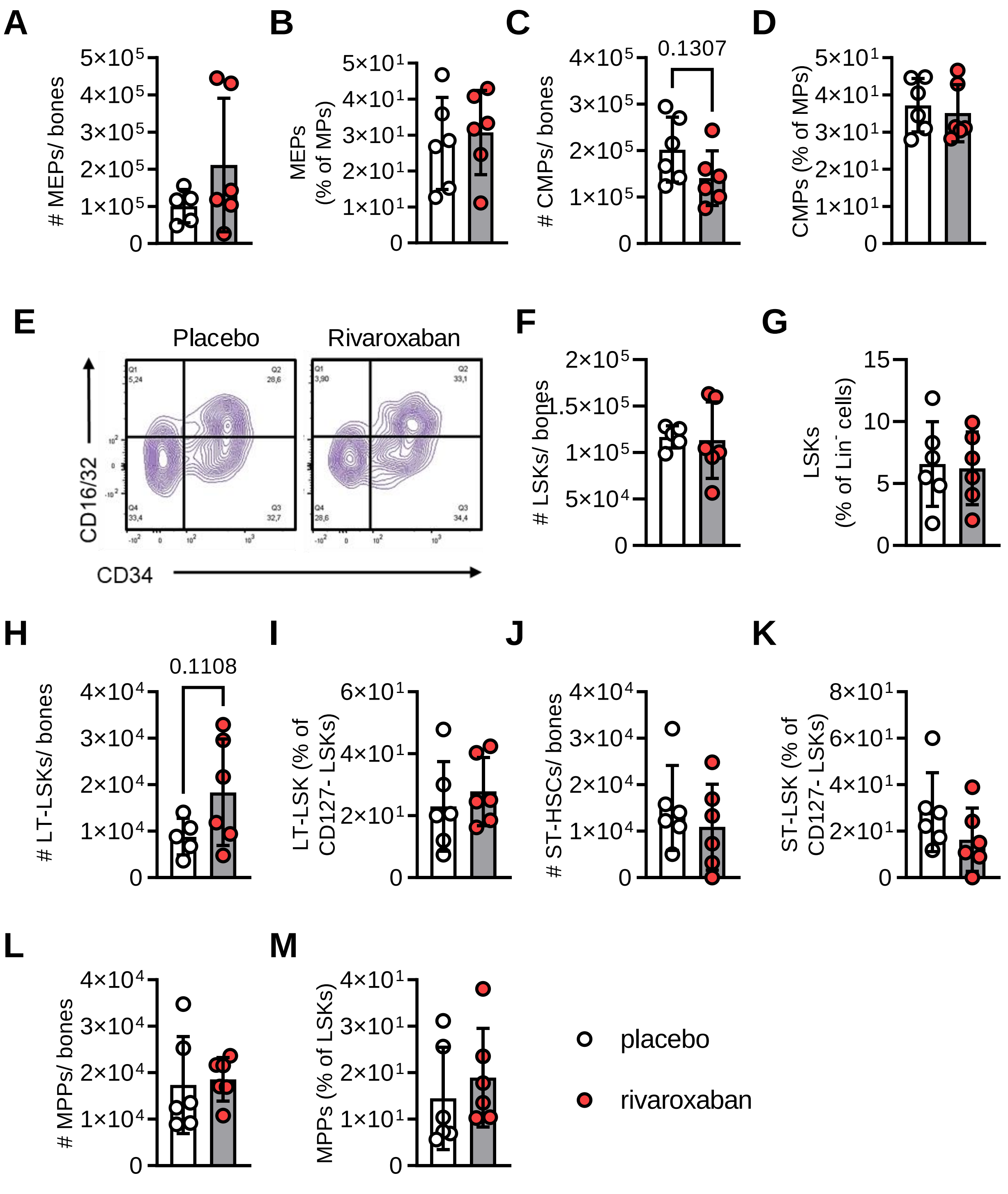

Figure S10. Treatment with rivaroxaban does not alter hematopoietic stem and progenitor cells in the bone marrow. Flow-cytometric analysis the bone marrow from tibiae and femora after 7 days of feeding with rivaroxaban or placebo. Quantification of **A-B,** megakaryocyte-erythrocyte progenitor cells (MEPs; $Lin^-Sca1^-c\text{-}KIT^+CD34^-CD16/32^-$); **C-D** common myleoid progenitor cells (CMPs; $Lin^-Sca1^-c\text{-}KIT^+CD34^+CD16/32^-$) and **E,** representative flow plot. Quantification of **F-G,** LSKs cells ((Lin)eage$^-$ $Sca1^+c\text{-}KIT^+$), **H-I** long term hematopoietic stem cells (LT-HSCs; $Lin^-Sca1^+c\text{-}KIT^+CD127^-CD34^-CD135^-$), **J-K** short term hematopoietic stem cells (ST-HSCs; $Lin^-Sca1^+c\text{-}KIT^+CD127^-CD34^+CD135^-$), **L-M** multipotent progenitor cells (MPPs; $Lin^-Sca1^+c\text{-}KIT^+CD127^-CD34^+CD135^+$). n = 5-6. Data represent mean ± SD; **P* < 0.05 vs. Placebo, two-tailed unpaired Student's t-test. # indicates absolute cell numbers.

Supplementary Figure S11

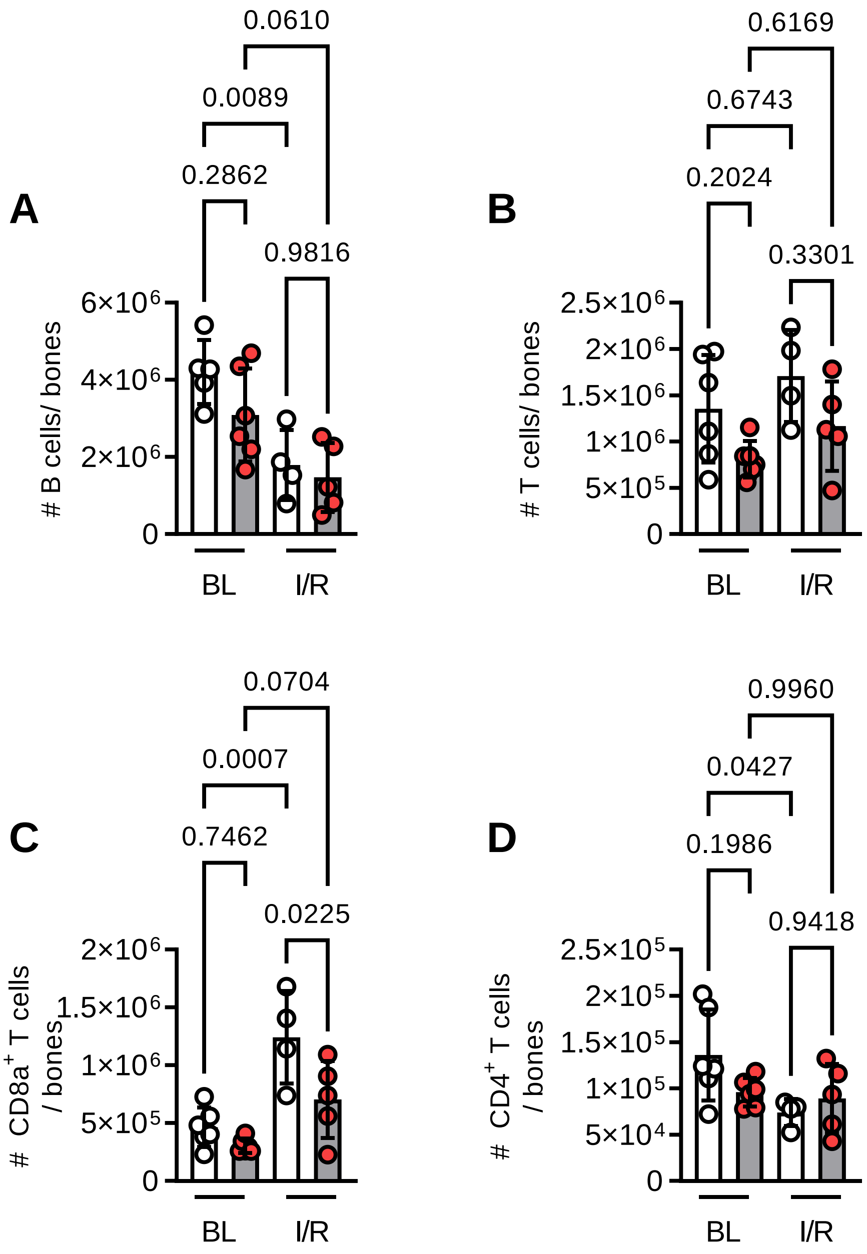

Figure S11. Bone marrow lymphocytes at steady state and 72 hours post I/R. C57BL6/J mice were treated with placebo or rivaroxaban for seven days and then subjected to cardiac I/R injury. Flow cytometric analysis of the bone marrow (BM) from tibiae and femora at baseline and 72 hours after I/R. **A,** B cells ($CD45^+CD19^+$), **B,** T cells ($CD45^+CD3^+$), **C,** $CD8a^+$ T cells ($CD45^+CD3^+CD8a^+$) , **D,** $CD4^+$ T cells ($CD45^+CD3^+CD4^+$) shown as absolute cell numbers (#); n = 4-6. Data represent mean ± SD; *P < 0.05 vs. placebo; Statistical significance was determined by Ordinary one-way ANOVA followed by Sidak post hoc test.

Supplementary Figure S12

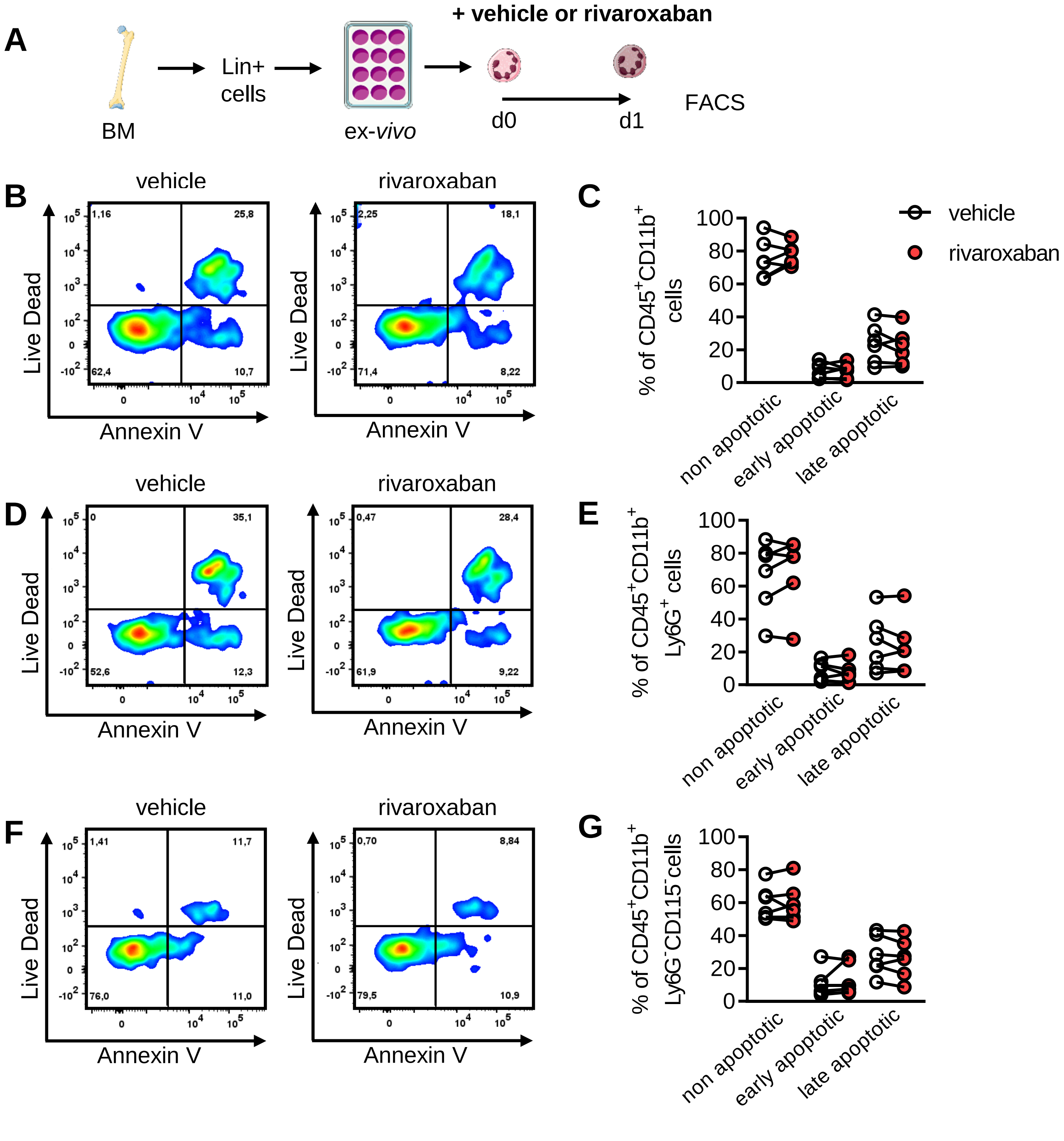

Figure S12. Apoptosis in bone marrow derived mature myeloid cells is not increased by rivaroxaban. A, experimental outline. Bone marrow aspirates from C57BL/6J mice were sorted for mature leukocytes (Lineage$^+$) using immunomagnetic bead separation. To test the impact of rivaroxaban on apoptosis, leukocytes were cultivated *ex-vivo* with vehicle or 10µM rivaroxaban for 24 hours followed by flow-cytometry. Combined Annexin V/Live Dead (LD) staining was used to determine non-apoptotic (Annexin$^-$LD$^-$), early apoptotic (Annexin$^+$LD$^-$) and late apoptotic (Annexin$^+$LD$^+$) cells. **B,** flow plots and **C,** quantification of myeloid leukocytes (CD45$^+$CD11b$^+$). **D,** flow plots and **E,** quantification of neutrophils (CD45$^+$CD11b$^+$Ly6G$^+$). **F,** flow plots and **G,** quantification of Ly6G$^-$CD115$^-$ myeloid cells (CD45$^+$CD11b$^+$Ly6G$^-$CD115$^-$); n= 6. Data represent symbols and lines.

Supplementary Figure S13

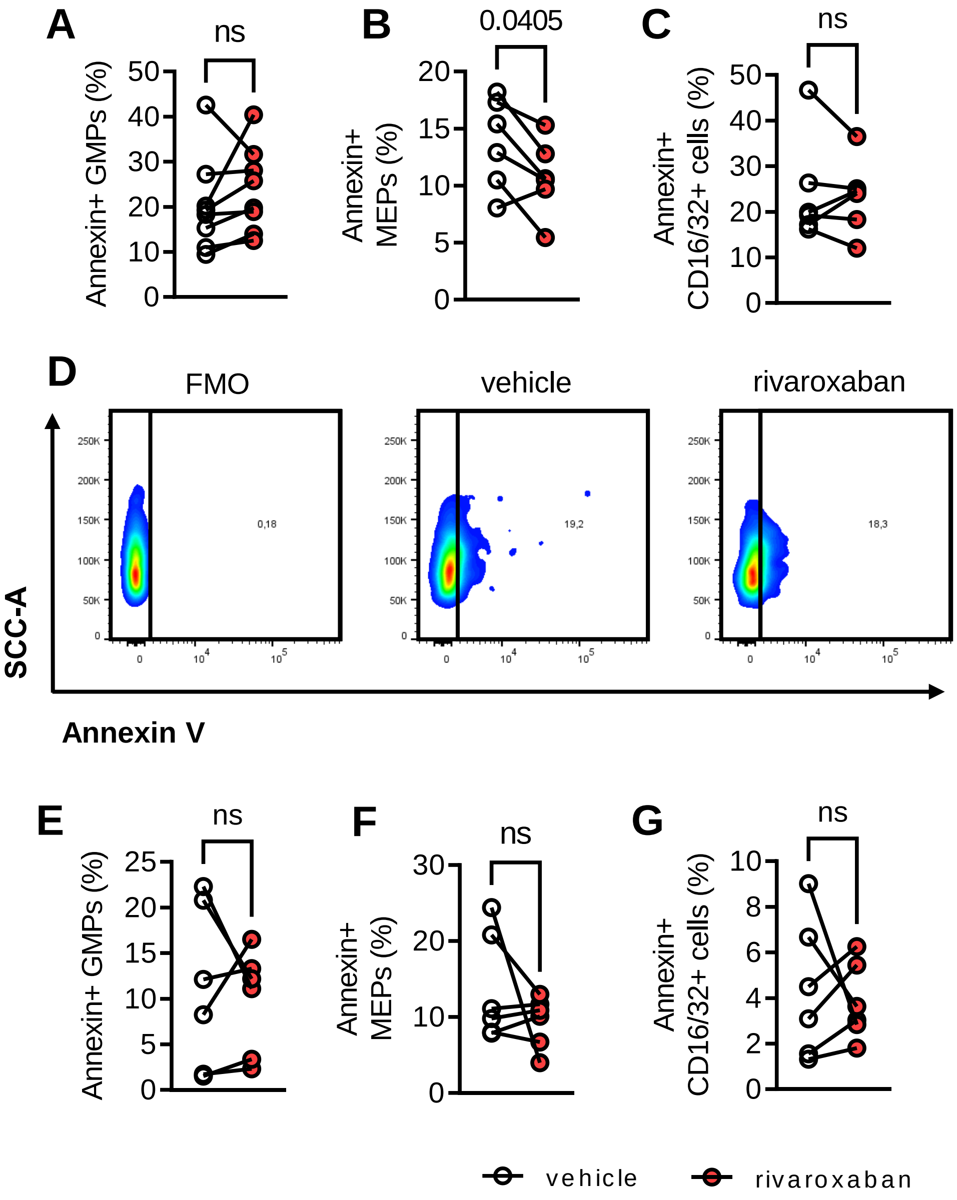

Figure S13. Impact of Rivaroxaban on apoptosis in myeloid progenitor cells after 24 and 72 hours of differentiation. Ex-vivo differentiation of bone marrow derived (Lin)eage- cells into myeloid progenitors (MPs). Cells were treated with vehicle or 10 µM rivaroxaban for 24 or 72 hours and harvested for flow-cytometric analyses. Annexin V staining was used to determine **A,** apoptotic granulocyte-monocyte progenitor cells (GMPs; $Lin^-Sca1^-c\text{-}KIT^+CD34^+CD16/32^+$), **B,** apoptotic megakaryocyte-erythrocyte progenitors (MEPs; $Lin^-Sca1^-c\text{-}KIT^+CD34^-CD16/32^-$), **C,** apoptotic $CD34^-CD16/32^+$ cells ($Lin^-Sca1^-c\text{-}KIT^+CD34^-CD16/32^+$) after 24 h of differentiation shown as percentages. **D,** Representative flow plots of apoptotic $CD34^-CD16/32^+$ cells. **E,** apoptotic granulocyte-monocyte progenitor cells (GMPs; $Lin^-Sca1^-c\text{-}KIT^+CD34^+CD16/32^+$), **F,** apoptotic megakaryocyte-erythrocyte progenitors (MEPs; $Lin^-Sca1^-c\text{-}KIT^+CD34^-CD16/32^-$), **G,** apoptotic $CD34^-CD16/32^+$ cells ($Lin^-Sca1^-c\text{-}KIT^+CD34^-CD16/32^+$) after 72 h of differentiation shown as percentages. Data represent symbols and lines. *$P < 0.05$, paired t-test. FMO indicates fluorescence minus one control.

Supplementary Figure S14

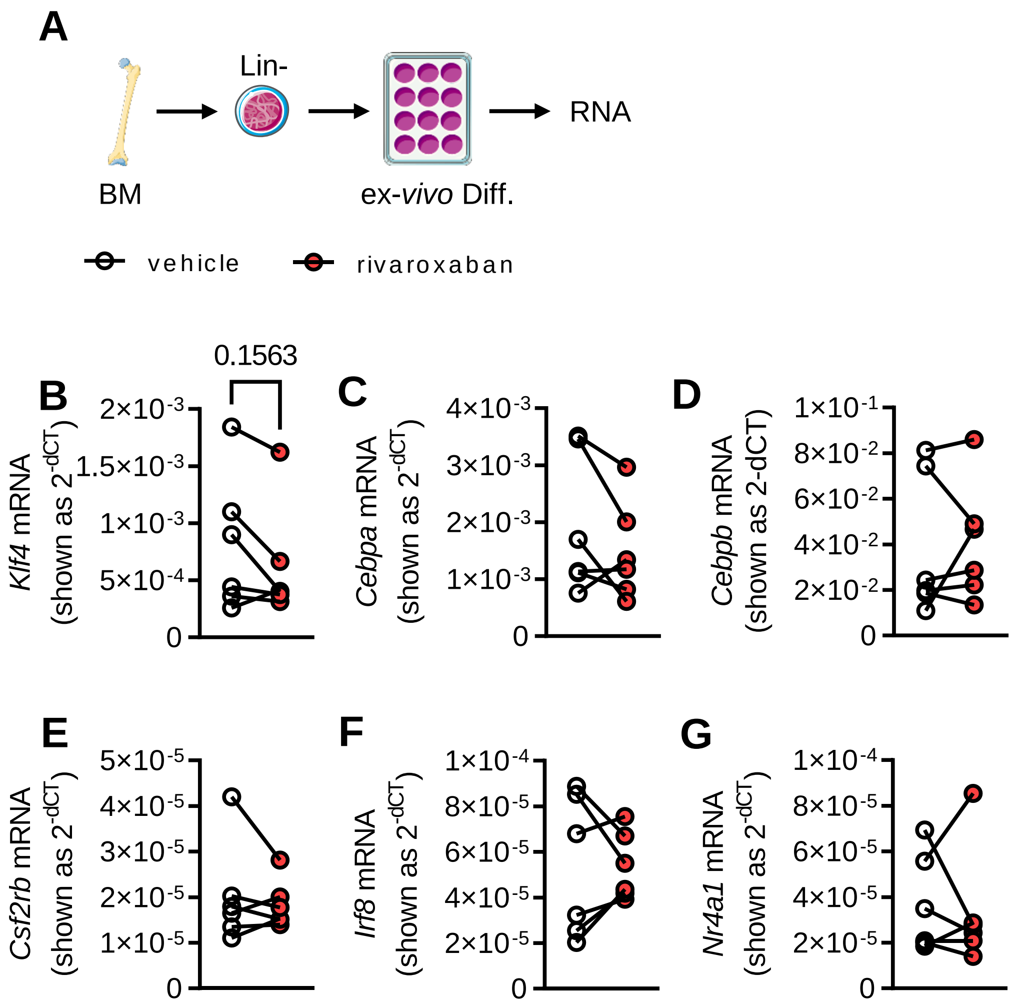

Figure S14. Rivaroxabans effect on transcription factors promoting GMP to myeloid leukocyte differentiation. A, experimental outline showing *ex-vivo* differentiation of bone marrow derived (Lin)eage^{-} cells into myeloid progenitors (MPs) (d3). Cells were treated with vehicle or 10 μM rivaroxaban and harvested for RNA isolation followed by semi-quantitative *realtime* PCR. n = 6. mRNA expression of **B,** krüppel like factor (*Klf4*), **C,** CCAT enhancer binding protein alpha (*Cebpa),* **D,** *Cebpb*, **E,** *Csf2rb,* **F,** interferon regulatory factor 8 (*Irf8)* and **G,** nuclear receptor 4A1 (*Nr4a1)* shown as $2^{\text{-delta (d) CT}}$. Data represent symbols and lines. **P* < 0.05, paired t-test **(B)** and Wilcoxon matched-pairs signed rank test **(C, F)**.

Supplementary Figure S15

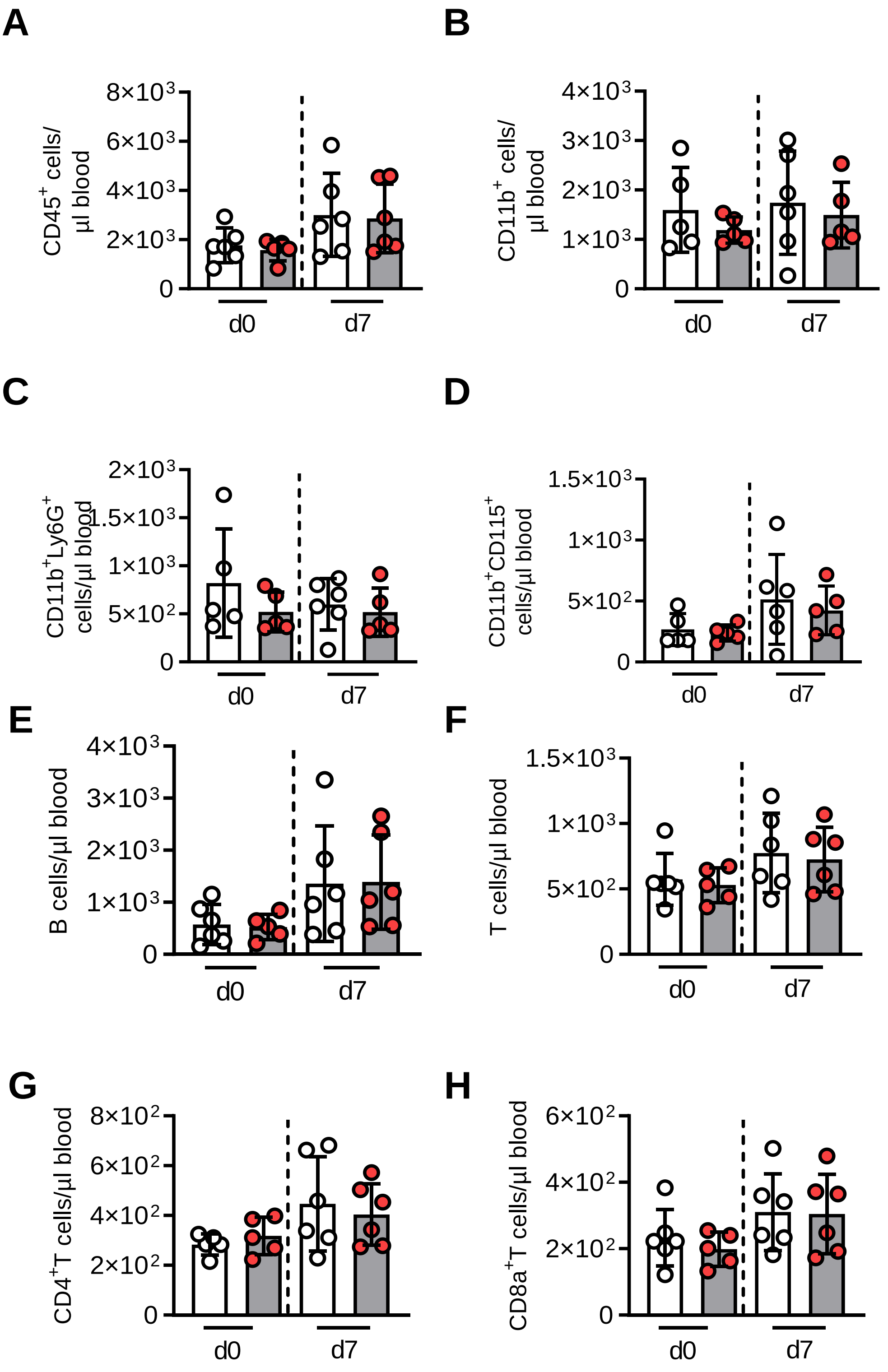

Figure RS15. Pretreatment with rivaroxaban does not alter the number of circulating leukocytes and lymphocytes. Flow cytometric analysis of circulating lymphocytes and myeloid leukocytes at baseline (d0) and after seven days of feeding (d7) with placebo or rivaroxaban. **A,** B cells ($CD45^+CD19^+$), **B,** T cells ($CD45^+CD3^+$), **C,** $CD4^+$ T cells ($CD45^+CD3^+CD4^+$), **D,** $CD8a^+$ T cells ($CD45^+CD3^+CD8a^+$), **E**, leukocytes ($CD45^+$), F, myeloid leukocytes ($CD11b^+$), **G,** neutrophils ($CD11b^+Ly6G^+$), **H,** monocytes ($CD11b^+CD115^+$) shown as cells per µl blood; n = 5-6. Data represent mean ± SD.

Supplementary Figure S16

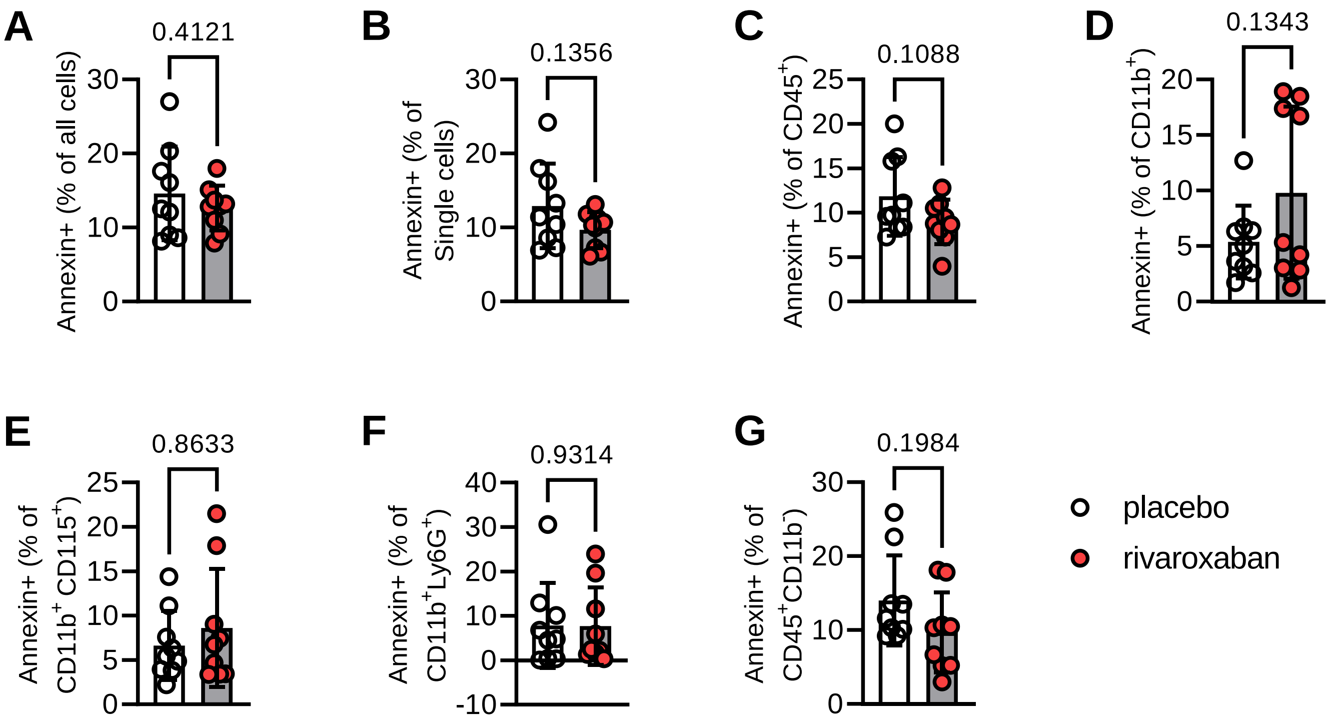

Figure S16. Apoptosis of circulating leukocytes at steady state. C57BL/6J mice were treated for seven days with rivaroxaban or placebo. Flow-cytometric analysis of Annexin+ cells in the bone marrow. **A**, all cells, **B**, Single cells, **C**, leukocytes ($CD45^+$), **D**, myeloid leukocytes ($CD45^+CD11b^+$), **E**, monocytes ($CD45^+$ $CD11b^+CD115^+$), **F**, neutrophiles, ($CD45^+CD11b^+Ly6G^+$), **G**, lymphocytes ($CD45^+$ $CD11b^-$) n = 9. Data represent mean ± SD, **P* < 0.05, ***P* < 0.01. Statistical significance was determined by unpaired t-test in A-D and Mann-Whitney test in E-G.

Supplementary Figure S17

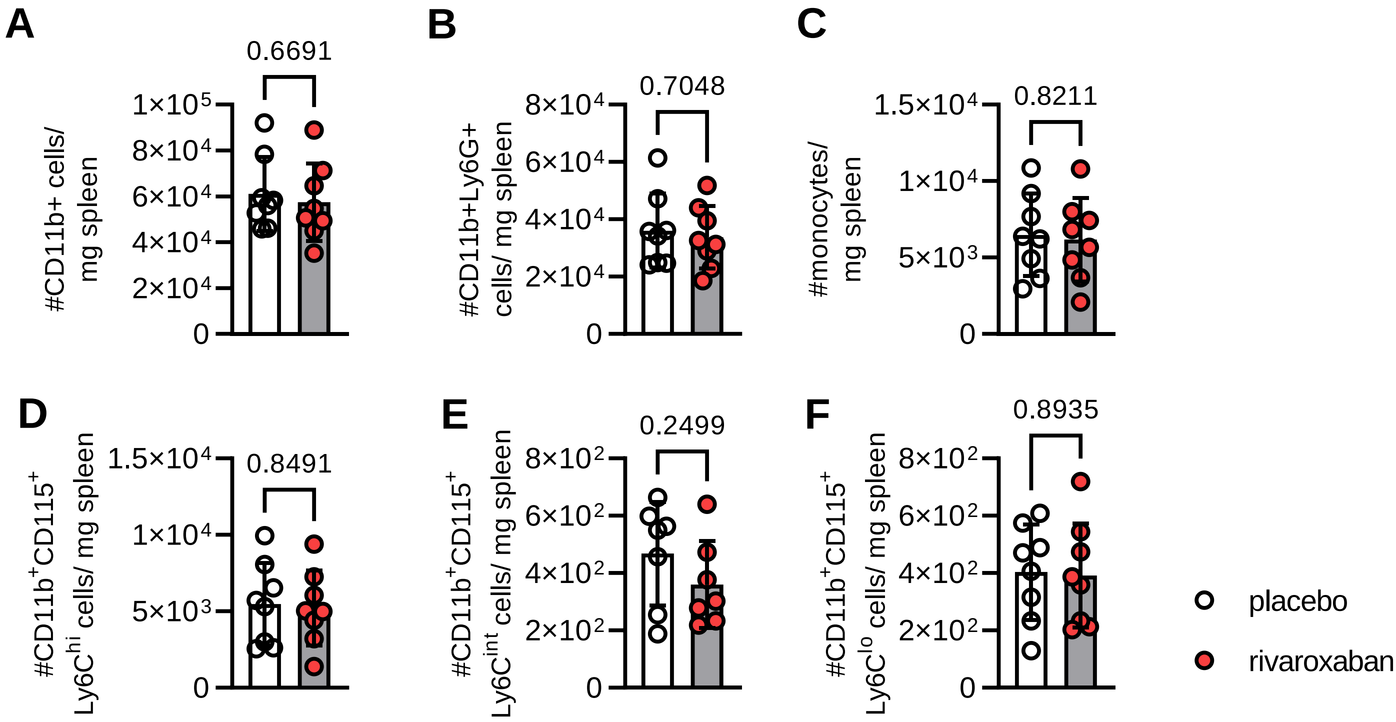

Figure S17. Splenic myeloid leukocytes at steady state. C57BL/6J mice were treated for seven days with rivaroxaban or placebo. Flow-cytometric analysis of myeloid leukocyte subsets in the spleen. **A,** myeloid leukocytes ($CD45^+CD11b^+$) **B,** neutrophils ($CD45^+CD11b^+Ly6G^+$), **C,** monocytes ($CD45^+CD11b^+CD115^+$), **D,** $Ly6C^{high}$ monocytes ($CD45^+CD11b^+CD115^+Ly6C^{high}$), **E,** $Ly6C^{int}$ monocytes ($CD45^+CD11b^+CD115^+Ly6C^{int}$), **F,** $Ly6C^{low}$ monocytes ($CD45^+CD11b^+CD115^+Ly6C^{low}$) shown as cells per mg tissue; n = 6. Data represent mean ± SD. ; *P < 0.05 vs. placebo; Statistical significance was determined by two-tailed unpaired Student's t-test.

Supplementary Figure S18

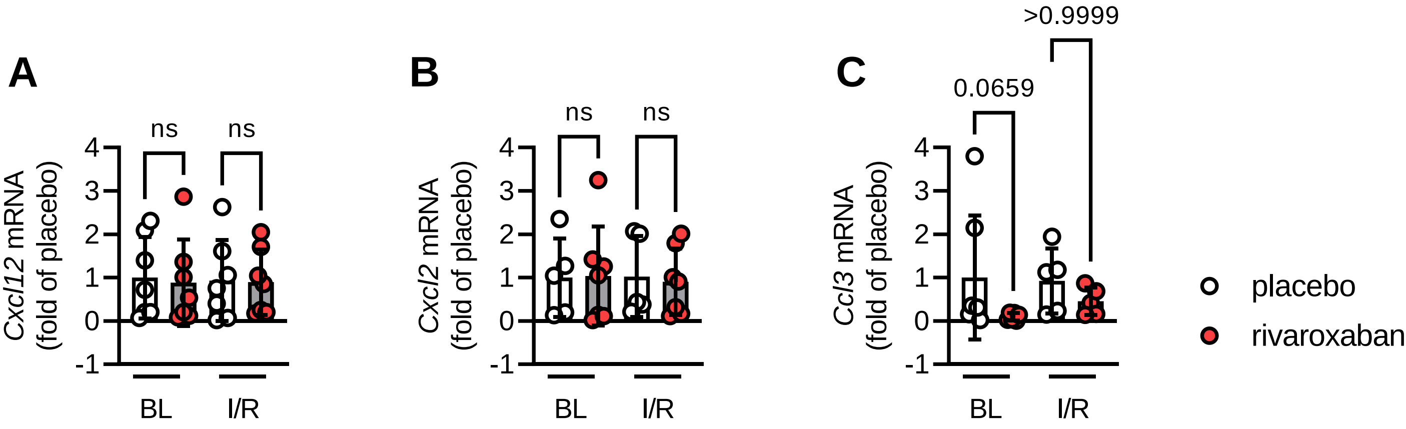

Figure S18. Effect of Rivaroxaban on retention and mobilizing factors in c-kit⁺ cells from the bone marrow. mRNA expression of **A**, C-X-C motif chemokine 12 (*Cxcl12*), **B**, C-X-C motif chemokine 2 (*Cxcl2)* and **C,** C-C motif chemokine ligand 3 (*Ccl3*) at steady state (BL) and 24h hours post cardiac ischemia and reperfusion (I/R). n=5-7. Data represent mean ± SD, $^*P < 0.05$. Statistical significance was determined by Ordinary one-way ANOVA followed by Sidak post hoc test in **A** and Kruskal-Wallis Test followed by Dunn´s multiple comparisons test in **B-C**.

Supplementary Figure S19

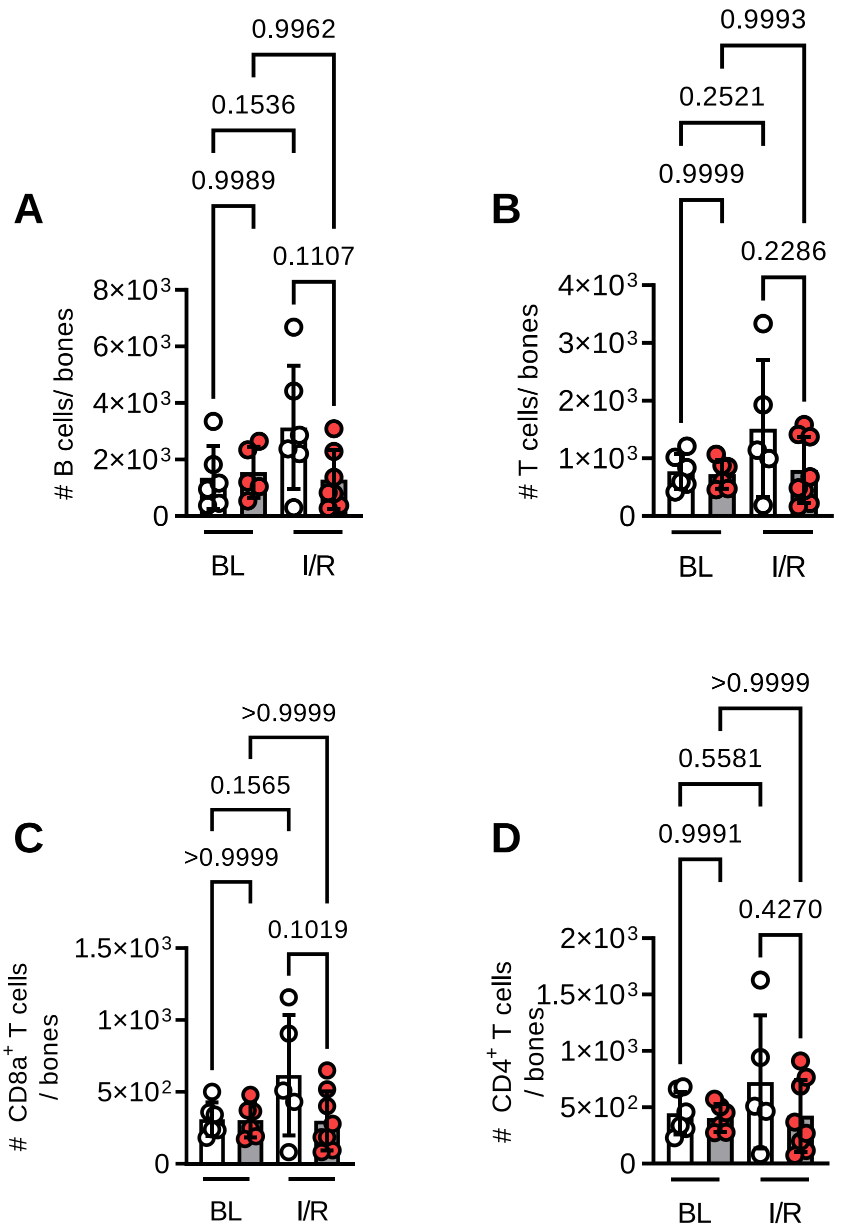

Figure S19. Circulating lymphocytes at steady state and 72 hours post I/R. C57BL6/J mice were treated with placebo or rivaroxaban for seven days and then subjected to cardiac I/R injury. Flow cytometric analysis of circulating lymphocytes at baseline and 72 hours after I/R. **A,** B cells ($CD45^+CD19^+$), **B,** T cells ($CD45^+CD3^+$), **C,** $CD8a^+$ T cells ($CD45^+CD3^+CD8a^+$) , **D,** $CD4^+$ T cells ($CD45^+CD3^+CD4^+$) shown as absolute cell numbers (#); n = 6. Data represent mean ± SD; **P* < 0.05 vs. placebo; Statistical significance was determined by Ordinary one-way ANOVA followed by Sidak post hoc test.

Supplementary Figure S20

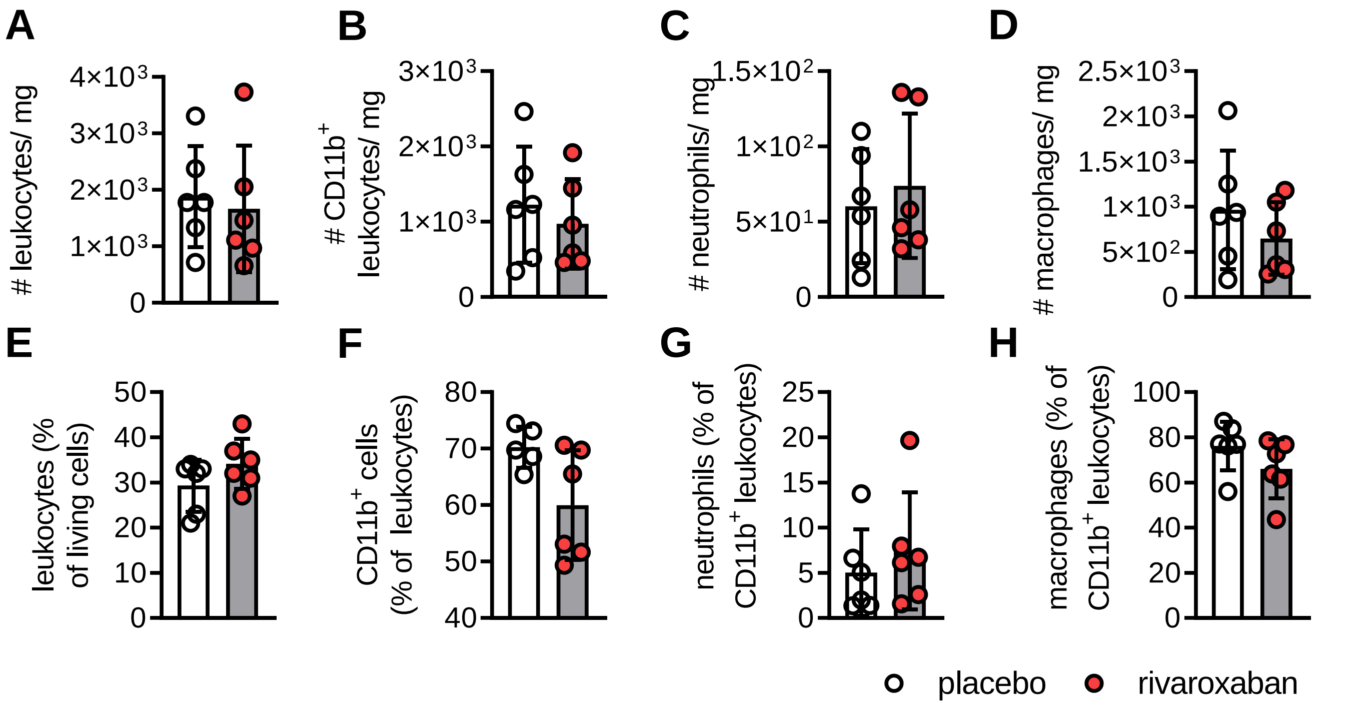

Figure S20. Pretreatment with Rivaroxaban does not change cardiac leukocyte subsets. Flow-cytometric analysis of cardiac immune cells after 7 days of feeding (d7) with rivaroxaban or placebo. **A,** leukocytes ($CD45^+$), **B,** myeloid leukocytes ($CD45^+CD11b^+$), **C,** neutrophils ($CD45^+CD11b^+Ly6G^+$) and **D,** macrophages ($CD45^+CD11b^+Ly6G^-F4/80^+$) shown as cells per mg heart and **E-H,** percentages. n = 5-6. Data represent mean ± SD.

Supplementary Figure S21

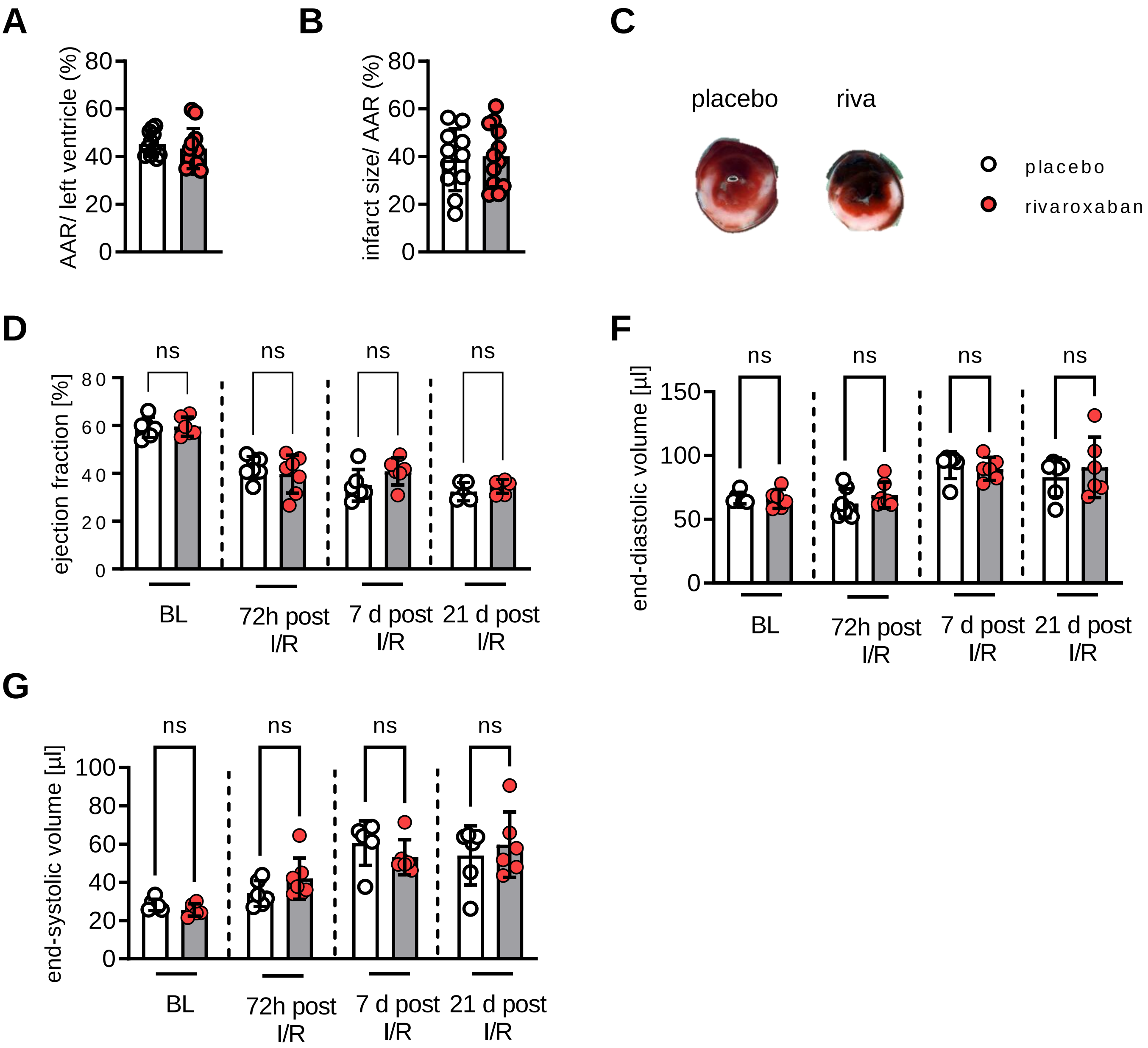

Figure S21. Cardiac Function post I/R. C57BL6/J mice were treated with placebo or rivaroxaban (riva) for seven days prior to I/R injury. Area at risk (AAR) and infarct size were determined by TTC-staining at 24 hours post I/R. **A,** area at risk (AAR), **B,** infarct size and **C,** representative TTC staining. n = 11-12. Hemodynamic function was assessed by echocardiography at baseline, 72 hours and 7 days post I/R and by MRI at 21 days post I/R. **D,** ejection fraction**, E**, end-systolic volume **F,** end-diastolic volume. n = 6-7. Data represent mean ± SD; Statistical significance was determined by two-tailed unpaired Student’s t-test. TTC = 2,3,5-Triphenyltetrazolium chloride.

Supplementary Table S1

Table S1: Descriptive statistics of rivaroxaban plasma concentrations [mg/l]

	1 day pre I/R 9 AM-0.6g/kg rivaroxaban	1 day pre I/R 9 AM-1.2g/kg rivaroxaban	1 day pre I/R 9 PM-0.6g/kg rivaroxaban	1 day pre I/R 9 PM-1.2g/kg rivaroxaban	1 day post I/R 9 AM-0.6g/kg rivaroxaban	1 day post I/R 9 AM-1.2g/kg rivaroxaban	1 day post I/R 9 PM-0.6g/kg rivaroxaban	1 day post I/R 9 PM-1.2g/kg rivaroxaban"
Number of values	3	3	3	3	3	3	3	2
Minimum	0.1260	0.4540	0.3480	1.080	0.03830	0.1050	0.05680	0.3320
Maximum	0.2560	0.9460	0.5520	1.770	0.3260	0.7120	0.1930	0.7120
Range	0.1300	0.4920	0.2040	0.6900	0.2877	0.6070	0.1362	0.3800
Mean	0.2060	0.7720	0.4223	1.520	0.1658	0.3820	0.1064	0.5220
Std. Deviation	0.07000	0.2758	0.1127	0.3822	0.1466	0.3070	0.07529	0.2687
Std. Error of Mean	0.04041	0.1592	0.06506	0.2207	0.08465	0.1772	0.04347	0.1900

Supplementary Table S2

Table S2: Antibodies used for flow cytometric analysis of heart, blood, spleen and bone marrow

Antibody	Clone	Cat.No.	Vendor	Concentration
Cardiac myeloid leukocytes				
CCR2-FITC	SA203G11	150608	Biolegend	1:25
F4/80-PE	BM8	123110	Biolegend	1:25
MHCII-PE/Cy7	M5/114.15.2	107630	Biolegend	1:25
CD11b-PE/Dazzle™	M1/70	101256	Biolegend	1:25
CD11c-APC	N418	117352	Biolegend	1:25
CD45-AF700	30-F11	103128	Biolegend	1:25
Ly-6C-APC/Cy7	HK1.4	128026	Biolegend	1:25
Ly-6G-PacBl	1A8	127612	Biolegend	1:25
LIVE/DEAD Fixable Aqua		L34965	Thermo Fisher Scientific	1:25
**Blood monocytes and neutrophils**				
CD115-APC	AFS98	17-1152-82	eBioscience	1:25
CD11b-PE	M1/70	101208	Biolegend	1:25
Ly-6C-AF488	HK1.4	128022	Biolegend	1:25
Ly-6G-PacBl	1A8	127612	Biolegend	1:25
**BM myeloid leukocytes**				
CD115-APC	AFS98	17-1152-82	eBioscience	1:25
CD11b-PE	M1/70	101208	Biolegend	1:25
Ly-6C-AF488	HK1.4	128022	Biolegend	1:25
Ly-6G-PacBl	1A8	127612	Biolegend	1:25
CD115-APC	AFS98	17-1152-82	eBioscience	1:25
**Splenic myeloid leukocytes**				
Ly6G-BV650	1A8	127641	Biolegend	1:80
CD45-PE	30-F11	103106	Biolegend	1:80
CD115-BV711	AFS98	135515	Biolegend	1:50
CD11b-PE/Dazzle	M1/70	101256	Biolegend	1:100
Ly6C APC/Cy7	HK1.4	128026	Biolegend	1:200

Supplementary Table S3

Table S3: Antibodies used for flow cytometric analysis of bone marrow

Antibody	Clone	Cat.No.	Vendor	Concentration
**BM stem and progenitor cells**				
Lineage-AF700	17A2; RB6-8C5; RA3-6B2; Ter-119; M1/70	79923	Biolegend	1:25
CD34-FITC	RAM34	11034181	eBioscience	1:25
CD34 PE-Dazzle	HM34 / SA376A4	128616	Biolegend	1:50
cKit(CD117)-PE	2B8	105808	Biolegend	1:25
CD16/32-PerCPCy5.5	93	101324	Biolegend	1:25
CD16/32-BV421	93	101331	Biolegend	1:50
IL7ra(CD127)-PE/Cy5	A7R34	135016	Biolegend	1:25
Sca-1-PECy7	D7	108114	Biolegend	1:25
CD135(Flt3)-APC	A2F10	135310	Biolegend	1:25
Ly6G-BV650	1A8	127641	Biolegend	1:80
CD45R(B220)-BV650	RA3-6B2	103241	Biolegend	1:80
CD90.2-BV650	30-H12	740443	BD Biosciences	1:80
NK1.1-BV650	PK136	108735	Biolegend	1:80
CD81-PerCPCy5.5	Eat-2	104912	Biolegend	1:80
CD11b-BV785	M1/70	101243	Biolegend	1:80
CD115-BV711	AFS98	135515	Biolegend	1:80
CD106-FITC	429	105706	Biolegend	1:200
Ly6C-APC/Cy7	HK1.4	128026	Biolegend	1:320
CD34-FITC	RAM34	11-0341-82	eBioscience	1:50

Supplementary Table S4

Table S4: Antibodies used for flow cytometric analysis of BM-derived cells

Antibody	Clone	Cat.No.	Vendor	Concentration
**Mature leukocyte marker**				
CD19-PacBl	6D5	115523	Biolegend	1:12.5
Ly6G-BV650	1A8	127641	Biolegend	1:12.5
CD45-PE	30-F11	103106	Biolegend	1:25
CD3-AF700	17A2	100216	Biolegend	1:50
CD8a-AF647	53-6.7	100724	Biolegend	1:50
CD115-BV711	AFS98	135515	Biolegend	1:50
F4/80-BV605	BM8	123133	Biolegend	1:50
CD11b-PE/Dazzle	M1/70	101256	Biolegend	1:100
CD4-FITC	RM4-5	MCD0401	LifeTechnologies	1:200
Ly6C-APC/Cy7	HK1.4	128026	Biolegend	1:200
**Hematopoietic stem and progenitor cell marker**				
Lineage-AF700	17A2; RB6-8C5; RA3-6B2; Ter-119; M1/70	79923; 133313	Biolegend	1:25
CD34 PE-Dazzle	HM34	128616	Biolegend	1:50
CD16/32-BV421	93	101331	Biolegend	1:50
CD48-PE	HM48-1	103405	Biolegend	1:50
c-Kit-PE/Cy7	2B8	105814	Biolegend	1:100
Sca-1-BV650	D7	108143	Biolegend	1:100
CD150-PE/Cy5	TC1512F12.2	115912	Biolegend	1:200

Supplementary Table S5

Table S5: Primer pairs used for qPCR experiments.

Gene symbol	Forward 5‘ – 3‘	Reverse 5‘ - 3‘
18s rRNA	GCAATTATTCCCCATGAACG	GGCCTCACTAAACCATCCAA
Il1b	GGATGAGGACATGAGCACCT	CGTCACACACCAGCAGGTTA
Tnf	CGAGTGACAAGCCTGTAGCC	AGCTGCTCCTCCACTTGGT
Spi1	AACAGATGCACGTCCTCGATAC	ACAAGGTTTGATAAGGGAAGCAC
Klf4	CTTTCCTGCCAGACCAGATG	GGTTTCTCGCCTGTGTGAGT
Cebpa	GATTCCGGTGTGGCCTGAAA	TCAAGGAGAAACCACCACGG
Cebpb	TTGATGCAATCCGGATCAAACG	CAGTTACACGTGTGTTGCGTC
Csf1r	TGGCCTTCCTTGCTTCTAAA	GATGTCCCTAGCCAGTCCAA
Csf2rb	AGGGCACATGAGAGCTGACT	CTATTTATCTGTGCCCCATGACATT
Irf8	GATCGAACAGATCGACAGCA	GCTGGTTCAGCTTTGTCTCC
Nr4a1	TCTGCCTTCCTGGAACTCTTCA	CAGGCCTGAGCAGAAGATGAG
Cxcl12	CATCAGTGACGGTAAACCAG	GCACAGTTTGGAGTGTTGAG
Cxcl2	GAAGTCATAGCCACTCTCAAGG	CCTCCTTTCCAGGTCAGTTAGC
Cxcl1	CCGAAGTCATAGCCACACTCAA	GCAGTCTGTCTTCTTTCTCCGTTA
Ccl3	CATCGTTGACTATTTTGAAACCAG	GCCGGTTTCTCTTAGTCAGGAA

Supplementary Table S5

Table S5: Baseline characteristics of patients with STEMI and pPCI

Baseline characteristics	antiplatelet control (n = 15)	rivaroxaban (n = 9)	p-Value
Sex, men % (n)	73 (11)	78 (7)	1.000
Age, years	58 ± 7	69 ± 11	0.030
Body mass index, kg/m^2	26 ± 2.9	27 ± 4.3	0.726
Diabetes mellitus, % (n)	13 (2)	22 (2)	0.615
Hypertension, % (n)	47 (7)	78 (7)	0.210
Dyslipidaemia, % (n)	47 (7)	56 (5)	1.000
Smoker, % (n)	80 (12)	67 (6)	0.635
Stroke / Transient ischemic attack, % (n)	13 (2)	11 (1)	1.000
Previous myocardial infarction, % (n)	13 (2)	22 (2)	0.615
SAPT, % (n)	0.0 (0)	22 (2)	0.042
Aspirin, % (n)	0.0 (0)	0.0 (0)	1.000
Clopidogrel, % (n)	0.0 (0)	22 (2)	0.0.42
DAPT, % (n)	100 (15)	78 (7)	0.130
Aspirin + Clopidogrel, % (n)	0.0 (0)	56 (5)	0.003
Aspirin + Prasugrel, % (n)	6.7 (1)	22 (2)	0.533
Aspirin + Ticagrelor, % (n)	93 (14)	0.0 (0)	<0.001
Statin, % (n)	93 (14)	89 (8)	1.000
ACE Inhibitor, % (n)	100 (15)	100 (9)	1.000
Beta Blockers, % (n)	80 (12)	89 (8)	1.000
Calcium Antagonist, % (n)	0.0 (0)	11 (1)	0.375
